# Effect of Neprilysin Inhibition on Alzheimer Disease Plasma Biomarkers: A Secondary Analysis of a Randomized Clinical Trial

**DOI:** 10.1001/jamaneurol.2023.4719

**Published:** 2023-12-18

**Authors:** Wagner S. Brum, Kieran F. Docherty, Nicholas J. Ashton, Henrik Zetterberg, Oskar Hansson, John J. V. McMurray, Kaj Blennow

**Affiliations:** 1 Department of Psychiatry and Neurochemistry, Institute of Neuroscience and Physiology, Sahlgrenska Academy at the University of Gothenburg, Mölndal, Sweden; 2 Graduate Program in Biological Sciences: Biochemistry, Universidade Federal do Rio Grande do Sul, Porto Alegre, Brazil; 3 British Heart Foundation Cardiovascular Research Centre, University of Glasgow, Glasgow, United Kingdom; 4 King’s College London, Institute of Psychiatry, Psychology and Neuroscience, Maurice Wohl Institute Clinical Neuroscience Institute, London, United Kingdom; 5 National Institute for Health and Care Research Biomedical Research Centre for Mental Health and Biomedical Research Unit for Dementia at South London and Maudsley NHS Foundation, London, United Kingdom; 6 Centre for Age-Related Medicine, Stavanger University Hospital, Stavanger, Norway; 7 Clinical Neurochemistry Laboratory, Sahlgrenska University Hospital, Mölndal, Sweden; 8 Department of Neurodegenerative Disease, Queen Square Institute of Neurology, University College London, London, United Kingdom; 9 UK Dementia Research Institute, University College London, London, United Kingdom; 10 Hong Kong Center for Neurodegenerative Diseases, Hong Kong, China; 11 Wisconsin Alzheimer’s Institute, School of Medicine and Public Health, University of Wisconsin, Madison, Wisconsin; 12 Clinical Memory Research Unit, Faculty of Medicine, Lund University, Lund, Sweden

## Abstract

This exploratory analysis of a randomized clinical trial evaluates the effect of neprilysin inhibition on Alzheimer disease blood biomarkers in patients with heart disease.

Amyloid-β (Aβ) accumulation is critical in Alzheimer disease (AD), and neprilysin is involved in physiologically clearing Aβ. Concerns exist regarding long-term use of sacubitril/valsartan, a neprilysin inhibitor and angiotensin receptor blocker used for heart failure, and its potential to increase AD risk. We evaluated neprilysin inhibition’s effect on AD blood biomarkers in patients with coronary heart disease.

## Methods

In a post hoc exploratory analysis of a 52-week randomized clinical trial (NCT03552575), we examined the effect of sacubitril/valsartan vs valsartan (ie, neprilysin inhibition) on AD blood biomarkers in patients with asymptomatic left ventricular systolic dysfunction late after myocardial infarction (eFigure and eMethods in Supplement 2). The primary analysis showed no significant results.^1^ Patients needed to be cognitively capable of independently adhering to the protocol (Supplement 1) throughout the study. The protocol was approved by the East of Scotland Research Ethics Committee. Patients provided informed consent. This study followed the CONSORT reporting guideline.

A 2-sided *P* < .05 was considered significant. Analyses were exploratory and not corrected for multiple testing. Participants were recruited between July 2018 and June 2019, with follow-up until June 2020. This data analysis was performed from November to December 2022 using R, version 4.1.1 (R Foundation for Statistical Computing).

## Results

**Figure 1.**
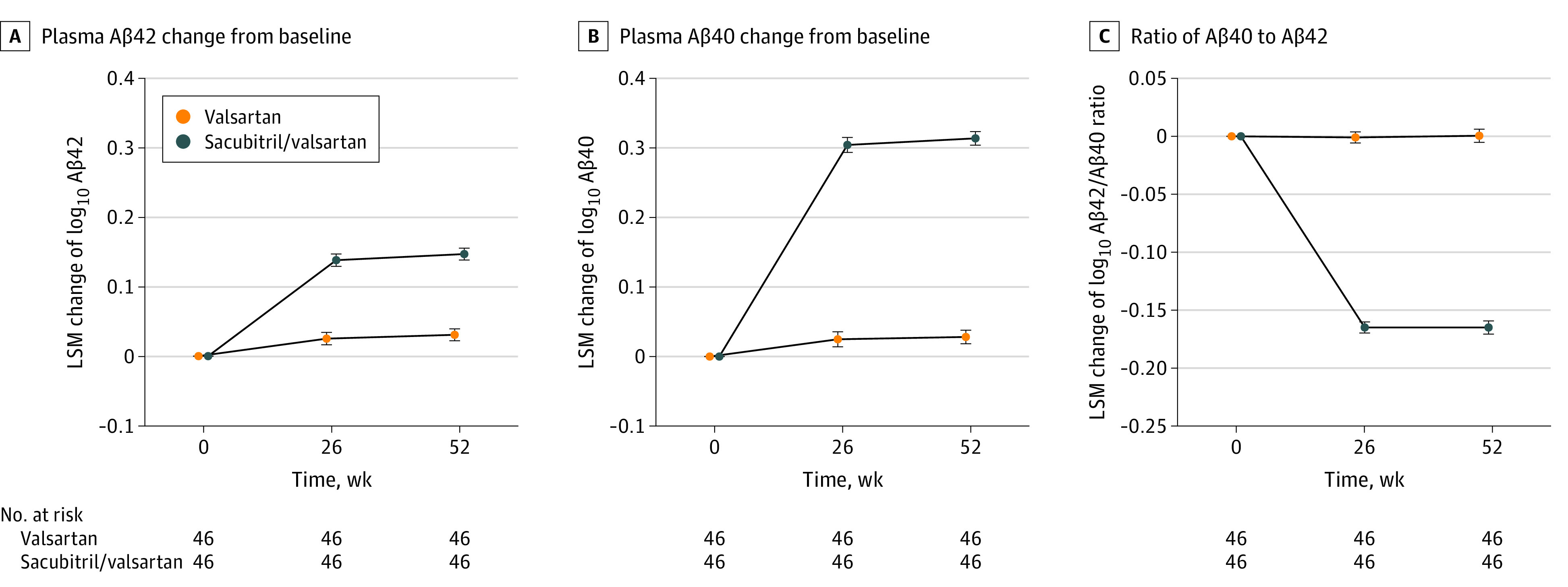
Changes in Amyloid-β (Aβ) Blood Biomarkers Following Sacubitril/Valsartan Treatment Plasma biomarker values were log_10_-transformed, with error bars indicating the SE of the adjusted between-group difference for each biomarker at each time point. Dotted lines represent the baseline as a reference. Relative differences in the sacubitril/valsartan group compared with the valsartan group was 31% for Aβ42, 93% for Aβ, and 32% for the ratio of Aβ42 to Aβ40 (all *P* < .001). LSM indicates least squares mean.

**Figure 2.**
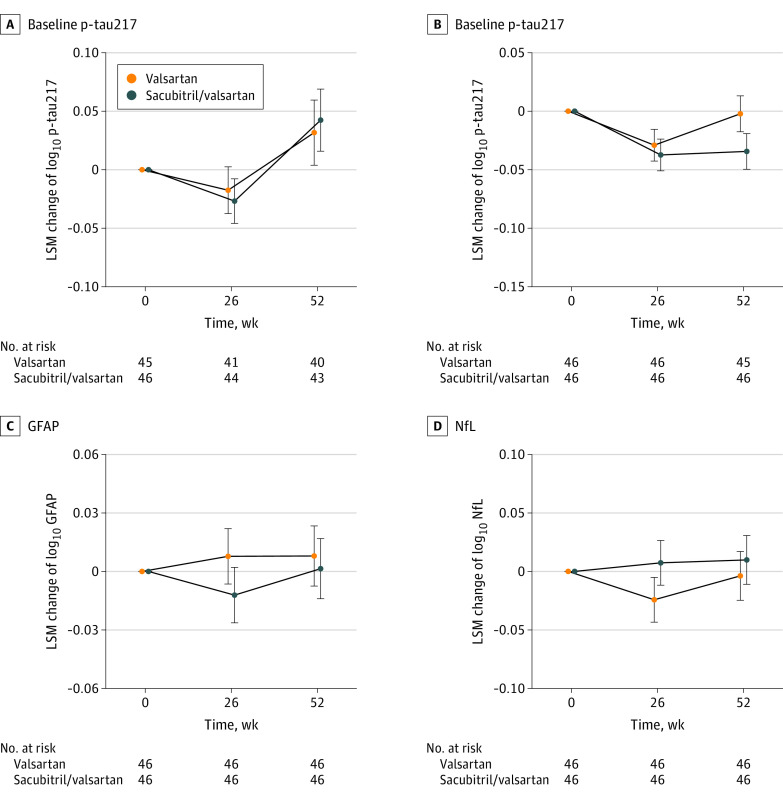
Changes in Other Blood Biomarkers Following Sacubitril/Valsartan Treatment Plasma biomarker values were log_10_-transformed, and error bars indicate the SE of the adjusted between-group difference for each biomarker at each time point. Dotted lines represent the baseline as a reference. No significant differences were observed for phosphorylated tau at threonine 181 (p-tau181) (2.5%; 95% CI, −13.9% to 22.1%; *P* = .78), phosphorylated tau at threonine 217 (p-tau217) (−7.1%; 95% CI, −15.8% to 2.4%; *P* = .14), glial fibrillary acidic protein (GFAP) (−1.5%; 95% CI, −10.8% to −8.8%; *P* = .77), or neurofilament light (NfL) (3.2%; 95% CI, −9.7% to 17.9%; *P* = .65).

Ninety-two patients (46 per group; mean [SD] age, 61.0 [10.3] years; 84 men [91.3%]; 8 women [8.7%]; 2 [2.8%] self-reporting as South Asian and 90 [97.8%] as White) were examined. At 26 weeks, the sacubitril/valsartan vs valsartan group showed significant increases from baseline in plasma Aβ42 and Aβ40, persisting at 52 weeks (Aβ42, 30.7% [95% CI, 23.7%-38.0%; *P* < .001]; Aβ40, 93.0% [95% CI, 81.3%-105.5%; *P* < .001]); plasma Aβ42/Aβ40 ratio significantly decreased at 26 weeks, persisting at 52 weeks (−31.7%; 95% CI, −34.1% to −29.1%; *P* < .001) (Figure 1). Notably, 3 female participants randomized to sacubitril/valsartan also experienced reductions in plasma Aβ42/Aβ40. No significant differences were observed for biomarkers of phosphorylated tau at threonine 217 (p-tau217) and 181, glial fibrillary acidic protein (GFAP), or neurofilament light (Figure 2).

## Discussion

These treatment-related increases in plasma Aβ42 and Aβ40 likely reflect reduced peripheral neprilysin activity because sacubitril effectively inhibits neprilysin without substantially crossing the blood-brain barrier.^2^ This pattern of Aβ42/Aβ40 reduction (increases in both peptides) differs from AD, wherein Aβ42/Aβ40 is reduced, reflecting pathologic decreases of Aβ42 and unchanged Aβ40 levels.^3^ Our findings align with a pharmacokinetic study showing that sacubitril/valsartan did not alter cerebrospinal fluid Aβ42 or Aβ40 levels in healthy volunteers but consistently increased plasma Aβ40 levels with a less sensitive immunoassay.^2^

Plasma p-tau biomarkers, particularly p-tau217, are known to associate with Aβ and tau pathologies and predict cognitive decline, while plasma GFAP associates with Aβ pathology, and neurofilament light with neuronal injury. While the absence of changes in these biomarkers (observed within a time frame in which p-tau217 and GFAP were already changed by anti-Aβ treatments^4^) is reassuring, treatment that substantially affected plasma Aβ did not affect other biomarkers.

Our study highlights sacubitril/valsartan’s potential to confound plasma Aβ42/Aβ40 tests for AD. In AD, this ratio is only reduced by 8% to 14%,^3^ while sacubitril/valsartan reduces it by approximately 30%. Given the frequent co-occurrence of heart disease and cognitive impairment and increasing clinical availability of plasma Aβ42/Aβ40 tests,^5^ results for patients receiving sacubitril/valsartan should be interpreted cautiously; treatment-related Aβ42/Aβ40 reductions may lead to false-positive results and misclassification of Aβ positivity as being AD. This drug interaction contraindication for an AD blood test underscores the importance of considering potential confounders, especially in patients with comorbidities, such as for p-tau and kidney disease,^6^ and suggests that a multibiomarker assessment may better control for factors affecting individual biomarker classes.

Limitations include the absence of cerebrospinal fluid and positron emission tomography biomarkers, which have been previously explored.^2^ Further studies with racial and ethnic diversity and between-sex balance are warranted. While not directly tested here, we do not consider sacubitril/valsartan-related increases in plasma Aβ to be concerning given sacubitril/valsartan’s successful clinical implementation over almost a decade.

## Notes

Supplement 1.
Trial Protocol

Supplement 2.
eMethods
**eFigure.** CONSORT Flow Diagram

Supplement 3.
Data Sharing Statement
