# Supplementary material for: Effect of Neprilysin Inhibition on Alzheimer Disease Plasma Biomarkers: A Secondary Analysis of a Randomized Clinical Trial
Source: JAMA Neurol. 2023 Dec 18;81(2):197–200. doi: 10.1001/jamaneurol.2023.4719 (PMC10728797; doi:10.1001/jamaneurol.2023.4719)
Supplement: Supplement 1. — Trial Protocol [file jamaneurol-e234719-s001.pdf]

The effects of sacubitril/valsartan compared to valsartan on left ventricular remodelling in patients with asymptomatic left ventricular systolic dysfunction after myocardial infarction: a randomised, double-blinded, active-comparator, cardiac-MR based trial

Protocol

Protocol Version:	6.0
Date:	27 03 2020
EudraCT Number:	2017-003460-13
REC Reference Number:	17/ES/0128
Sponsor's Protocol Number:	GN16CA007
Sponsors:	NHS Greater Glasgow & Clyde and The University of Glasgow
Funder:	BHF Project Grant no. PG/17/23/32850<br>IMP supplied by Novartis Pharmaceuticals UK Ltd.

This study will be performed according to the Research Governance Framework for Health and Community Care (Second edition, 2006) and The Medicines for Human Use (Clinical Trials) Regulations, 2004 SI 2004:1031 (as amended) and World Medical Association Declaration of Helsinki Ethical Principles for Medical Research Involving Human Subjects 1964 (as amended).

Contacts

Chief Investigator

Professor John J.V. McMurray
Professor of Cardiology
Institute of Cardiovascular and Medical Sciences
BHF Glasgow Cardiovascular Research Centre
126 University Place
University of Glasgow
Glasgow, G12 8TA

Co-investigators

Professor Mark C. Petrie
Consultant Cardiologist
Glasgow Royal Infirmary/Golden Jubilee National Hospital
BHF Glasgow Cardiovascular Research Centre
126 University Place
Glasgow, G12 8TA

Dr. Ross T. Campbell
Specialist Registrar/Clinical Lecturer
BHF Glasgow Cardiovascular Research Centre
126 University Place
Glasgow, G12 8TA

Dr. Kieran F. Docherty
Specialist Registrar/Clinical Research Fellow
BHF Glasgow Cardiovascular Research Centre
126 University Place
Glasgow, G12 8TA

Trial Statistician

Dr. Alex McConnachie
Assistant Director of Biostatistics
Robertson Centre for Biostatistics
Boyd Orr Building, Level 11
University of Glasgow
Glasgow, G12 8QQ

Chair of the Trial Steering Committee

Professor Iain Squire
Professor of Cardiovascular Medicine
University of Leicester
Glenfield Hospital
Leicester
LE3 9QP

Pharmacovigilance

Dr. Marc Jones
Pharmacovigilance and Safety Manager
NHS Greater Glasgow & Clyde
Research and Development Management Office
West Glasgow Ambulatory Care Hospital
Dalnair Street
Glasgow, G3 8SW

IMP Management

Dr. Elizabeth Douglas
Senior Pharmacist, Clinical Trials
NHS Greater Glasgow & Clyde
Research and Development Management Office
West Glasgow Ambulatory Care Hospital
Dalnair Street
Glasgow, G3 8SW

Project Manager

Dr. Katriona Brooksbank
Clinical Trials Manager
Institute of Cardiovascular and Medical Sciences
BHF Glasgow Cardiovascular Research Centre
126 University Place
University of Glasgow
Glasgow, G12 8TA

Sponsor

This clinical trial is sponsored by NHS Greater Glasgow & Clyde and the University of Glasgow

Sponsor's representative

Dr. Maureen Travers
Academic Research Coordinator
NHS Greater Glasgow & Clyde
Research and Development Management Office
West Glasgow Ambulatory Care Hospital
Dalnair Street
Glasgow, G3 8SW

Funding Body

BHF Project Grant no. PG/17/23/32850
IMP provided by Novartis Pharmaceuticals UK Ltd.

Table of Contents

Contacts **2**

Table of Contents **4**

Abbreviations **6**

Study Synopsis **8**

Study Flow Chart **10**

1 Introduction **11**
- **1.1 Background** **11**
- **1.2 Study Rationale – hypothesis** **12**

2 Study Objectives **13**
- **2.1 Primary objective** **13**
- **2.2 Secondary objectives** **13**

3 Endpoints **13**
- **3.1 Primary Endpoints** **13**
- **3.2 Secondary Endpoints** **13**
- **3.3 Tertiary Endpoints** **13**

4 Investigational Plan **14**
- **4.1 Study Design** **14**
- **4.2 Rationale of key elements of the study design** **16**

5 Patient Population **17**
- **5.1 Inclusion Criteria** **18**
- **5.2 Exclusion Criteria** **18**
- **5.3 Women of child bearing potential** **18**
- **5.4 Males whose partner could become pregnant** **19**
- **5.5 Pregnancy** **19**

6 Study Treatment **19**
- **6.1 Investigational and control drugs** **19**
- **6.2 Study Drug storage and supply** **20**
- **6.3 Drug accountability requirements** **21**
- **6.4 Treatment arms** **21**
- **6.5 Permitted dose adjustments and discontinuation of study treatment** **21**
- **6.6 Concomitant medications** **23**
- **6.7 Identification of participants and treatment assignment** **24**
- **6.8 Monitoring of potential side effects** **26**
  - 6.8.1 Management of nephrotoxicity 26
  - 6.8.2 Management of hyperkalaemia 27
  - 6.8.3 Management of hypotension 28
  - 6.8.4 Management of angioedema 28
- **6.9 Withdrawal of treatment** **28**
- **6.10 Unblinding procedure** **28**

7 Assessment and management of risk **29**

8 Visit schedule and assessments **30**
- **8.1 Screening** **30**
  - 8.1.1 Echocardiography protocol 31
- **8.2 Pre-randomisation investigations** **31**
  - 8.2.1 Biochemical and hormonal measurements 31
  - 8.2.2 CMR protocol 32
- **8.3 26-week visit** **32**

8.4 52-week visit 32
8.5 Definition of end of trial 33

9 Assessment, samples and data collection 33

10 Pharmacovigilance 35
10.1 Definitions 35
10.2 Recording and reporting of Adverse Events 36
10.3 Assessment of adverse events 36
10.4 Reporting to sponsor (Pharmacovigilance Office) 37
10.5 Reporting to the MHRA and MREC 38
10.6 Responsibilities 38
10.7 Pregnancy reporting 39
10.8 Development safety update reports 39

11 Statistics and data analysis plan 39
11.1 Sample size 39
11.2 Planned recruitment rate 40
11.3 Statistical analysis 40

12 Data handling 40
12.1 Randomisation 40
12.2 Case Report Forms 41
12.3 Record Retention 41

13 Trial management 41
13.1 Trial steering committee (TSC) 41

14 Study monitoring and auditing 41

15 Ethical considerations 41

16 Statement of indemnity 42

17 Funding 42

18 Sponsor responsibilities 42

19 Dissemination of data 42

20 References 43

Appendix 1: Clinically notable laboratory values 47

Appendix 2: MRI Information Leaflet 48
Appendix 3: Patient global assessment questionnaire 50

Tables and Figures

Table 4-1 Total daily doses of commonly used ACE inhibitors or ARBs corresponding to ramipril 2.5mg BD (dose level 2 of study drug) 14
Table 4-2 Study drug dose levels during treatment period 15
Table 4-3 Safety monitoring criteria that must be met for dose up-titration 16
Table 6-1 Prohibited medications 23
Table 8-1 Schedule of Assessments 34
Table 10-1 Pharmacovigilance Definitions 35

Figure 4-1 Study drug initiation and up-titration 15

Abbreviations

ACE: Angiotensin Converting Enzyme
AE: Adverse Event
ANP: Atrial Natriuretic Peptide
AMI: Acute Myocardial Infarction
AR: Adverse Reaction
ARB: Angiotensin Receptor Blocker
ARNI: Angiotensin Receptor Neprilysin Inhibitor
AUC: Area Under the Curve
BHF: British Heart Foundation
BD: *Bis in Die* - twice daily
BNP: Brain Natriuretic Peptide
BSA: Body Surface Area
CCF: Congestive Cardiac Failure
CCU: Coronary Care Unit
cGMP: cyclic Guanosine Monophosphate
CI: Chief Investigator
CMR: Cardiac Magnetic Resonance imaging
CNORIS: Clinical Negligence and Other Risks Indemnity Scheme
CNP: C-type Natriuretic Peptide
COX-2: Cyclo-oxygenase-2
CRF: Case Report Form
CRN: Clinical Research Network
CRP: C-Reactive Protein
CTIMP: Clinical Trial of Investigational Medical Product
CTU: Clinical Trials Unit
DSUR: Development Safety Update Report
ECG: Electrocardiograph
EF: Ejection Fraction
eGFR: estimated Glomerular Filtration Rate
FSH: Follicle Stimulating Hormone
GCP: Good Clinical Practice
GCRC: Glasgow Cardiovascular Research Centre
GG&C: Greater Glasgow and Clyde
GP: General Practitioner
GRI: Glasgow Royal Infirmary
HF-REF: Heart Failure with Reduced Ejection Fraction
HF: Heart Failure
hsTnT: high sensitivity Troponin-T
ICFs: Informed Consent Forms
ICH: International Conference on Harmonisation
ICTP: carboxy-terminal telopeptide of type I procollagen
IMP: Investigational Medical Product
IVRS: Interactive Voice Response Service
LBBB: Left Bundle Branch Block
LGE: Late Gadolinium Enhancement
LV: Left Ventricle
LVESV: Left Ventricular End Systolic Volume
LVSD: Left ventricular Systolic Dysfunction

MHRA: Medicines and Healthcare Regulatory Authority
MI: Myocardial Infarction
MMPs: matrix metalloproteinases
MR: Magnetic Resonance
MRA: Mineralocorticoid Receptor Antagonist
MREC: Medical Research Ethics Committee
NEP: Neprilysin
NSAIDs: Non-steroidal anti-inflammatory drugs
NSTEMI: Non ST Elevation Myocardial Infarction
NT-proBNP: N-Terminal pro BNP
NYHA: New York Heart Association
PGA: Patient Global Assessment
PICP: carboxy-terminal propeptide of type I procollagen
PIIINP: amino-terminal propeptide of type III procollagen
PV: Pharmacovigilance
QEUH: Queen Elizabeth University Hospital
RAAS: Renin Angiotensin Aldosterone System
RAH: Royal Alexandra Hospital
REC: Research Ethics Committee
SAE: Serious Adverse Event
SAR: Serious Adverse Reaction
SBP: Systolic Blood Pressure
SDV: Source Data Verification
SmPC: Summary of Product Characteristics
STEMI: ST Elevation Myocardial Infarction
SUSAR: Suspected Unexpected Serious Adverse Reaction
TIMP: Tissue Inhibitors of Metalloproteinases
TMG: Trial Management Group
TSC: Trial Steering Committee
TTE: Transthoracic Echocardiogram
U&Es: Urea and Electrolytes
ULN: Upper Limit of Normal range
WoCBP: Women of Child Bearing Potential

Study Synopsis

Title of study	The effects of sacubitril/valsartan compared to valsartan on left ventricular remodelling in asymptomatic left ventricular systolic dysfunction after myocardial infarction: a randomised, double-blinded, active-comparator, cardiac-MR based trial
Short title	RECOVER-LV
Study centre	Queen Elizabeth University Hospital Clinical Research Facility
Primary endpoint	Change in indexed left ventricular end-systolic volume (LVESVI), from baseline to 12 months (- 3months or + 6months) measured using cardiac MR.
Secondary endpoints	• Changes in other CMR based metrics of LV remodeling (LVEDVI, LVEF, LV mass) from baseline to 12 months(- 3months or + 6months)<br>• Changes in hsTnI from baseline to 12 months (- 3months or + 6months)<br>• Changes in NT-proBNP from baseline to 12 months (- 3months or + 6months)<br>• Change in patient well-being as assessed by a patient global assessment questionnaire.
Tertiary endpoints	• Changes in biomarkers of LV remodelling (sST2, Galectin 3, TIMP-1, MMP-9, Type III Procollagen Peptide and GDF-15) from baseline to 12 months(- 3months or + 6months)<br>• Changes in neurohormonal levels (BNP, MR-proANP, C-terminal ANP, CNP, MR-proADM, cGMP, endothelin-1, neprilysin antigen, renin and aldosterone) from baseline to 12 months (- 3months or + 6months)
Rationale	Prior to reperfusion therapy, the major therapeutic breakthrough in myocardial infarction was the demonstration that ACE inhibitors or ARBs, given to prevent adverse “remodelling” (progressive dilatation and decline in systolic function) in high risk patients, reduced the likelihood of developing heart failure and the risk of death. The neurohumoral systems which are activated in patients after myocardial infarction (and in heart failure) are not all harmful and some endogenous systems may be protective. The best recognised of these is the natriuretic peptide system. A- and B-type natriuretic peptides are secreted by the heart when it is stressed and these peptides promote vasodilation (reducing left ventricular wall stress), stimulate renal sodium and water excretion (i.e. antagonising the retention of salt and water characterising heart failure) and inhibit pathological growth i.e. hypertrophy and fibrosis (key components of the adverse left ventricular remodelling that occurs after infarction and in heart failure).The augmentation of plasma levels of endogenous natriuretic peptides can be achieved through inhibition of neutral endopeptidase, also known as neprilysin (NEP), which is responsible for the breakdown of natriuretic peptides. Recently, the addition of neprilysin inhibition to blockade of the RAAS (using sacubitril/valsartan), compared with RAAS blockade alone, reduced the risk of heart failure hospitalisation and death in patients with HF-REF. These exciting findings may lead to a new approach to the treatment of heart failure, with an angiotensin receptor neprilysin inhibitor (ARNI) replacing an ACE inhibitor as one of the fundamental treatments for this condition. We believe that the same approach may be beneficial in high-risk survivors of myocardial infarction. Recently, sacubitril/valsartan was shown to ameliorate adverse left ventricular remodelling in an experimental model of acute myocardial infarction. The objective of the present proposal is to gather “proof-of-concept”, mechanistic, evidence in humans to support adoption of this new approach in patients at high risk after myocardial infarction as a result of residual left ventricular systolic dysfunction.
Methodology	Prospective, randomised, active-comparator, double-blinded study.

Sample size	100
Screening	Patients with EF ≤40% measured by trans-thoracic echocardiography post myocardial infarction without evidence of clinical heart failure.
Inclusion criteria	• Acute myocardial infarction (AMI) at least 3 months prior to recruitment<br>• Left ventricular ejection ≤40% as measured by transthoracic echocardiography<br>• Ability to provide written, informed consent<br>• Age ≥18 years<br>• Tolerance of a minimum dose of ACE inhibitor/ARB (ramipril 2.5mg BD or equivalent)<br>• Treatment with a beta-blocker unless not tolerated or contraindicated.
Exclusion criteria	• Contraindication to CMR (ferrous prosthesis, implantable cardiac device or severe claustrophobia)<br>• Clinical and/or radiological heart failure (NYHA≥2)<br>• Symptomatic hypotension and/or systolic blood pressure <100mmHg<br>• eGFR < 30 mL/min/1.73m^2 and/or serum potassium >5.2mmol/L<br>• Persistent/permanent atrial fibrillation<br>• History of AMI within last 3 months<br>• History of hypersensitivity or allergy to ACE-inhibitors/ARB<br>• History of angioedema<br>• Known hypersensitivity to the active study drug substances, contrast media or any of the excipients<br>• Obesity (where body girth exceeds MRI scanner diameter)<br>• Pregnancy, planning pregnancy, or breast feeding<br>• Inability to give informed consent or comply with study protocol<br>• Evidence of hepatic disease as determined by any one of the following: AST or ALT values exceeding 2 x ULN at Visit 1, history of hepatic encephalopathy, history of oesophageal varices, or history of portacaval shunt<br>• History of biliary cirrhosis and cholestasis<br>• Active treatment with cholestyramine or colestipol resins<br>• Active treatment with lithium or direct renin inhibitor<br>• Participation in another intervention study involving a drug or device within the past 90 days (co-enrolment in observational studies is permitted)
Study drugs	Patients will be randomised in a 1:1 fashion to sacubitril/valsartan (target dose 97mg/103mg BD) or valsartan (target dose 160mg BD) with matched placebo.<br><br>• *Sacubitril/valsartan:* 24mg/26mg (dose level 1), 49mg/51mg (dose level 2) and 97mg/103mg (dose level 3) twice daily.<br>• *Valsartan:* 40mg (dose level 1), 80mg (dose level 2) and 160mg (dose level 3) twice daily.
Duration of treatment:	12 months (- 3months or + 6months in response to the COVID-19 pandemic)
Statistical analysis	The primary outcome will be compared between randomised groups using a linear regression model adjusted for baseline LVESVI value, and whether or not taking diuretics at baseline. Similar methods will be used for other outcomes. The Robertson Centre for Biostatistics will manage and analyse trial data. All statistical analyses will be conducted according to a Statistical Analysis Plan, which will be authored by the Trial Statistician and agreed by the Trial Steering Committee prior to unblinding of randomised groups.

Study Flow Chart

Version 6.0 – In response to COVID-19 the treatment time and study windrow for the week 52 visit have been altered (-3months + 6months) in order to be able to obtain the primary outcome for this clinical trial and ensure patient participation has been worthwhile

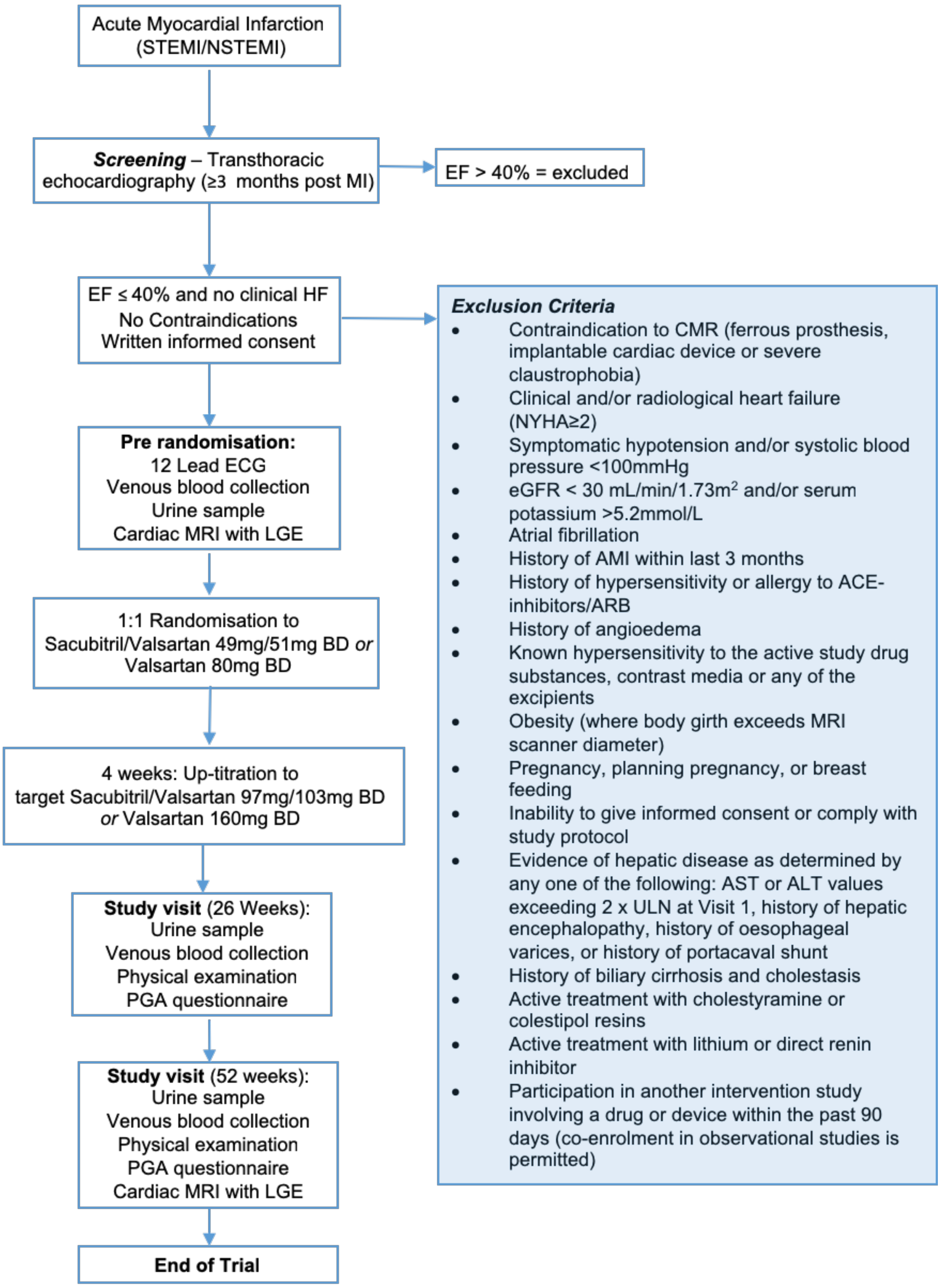

1 Introduction

1.1 Background

The widespread adoption of coronary reperfusion therapy (initially with thrombolysis and latterly with percutaneous intervention) has reduced the extent of left ventricular damage sustained during acute myocardial infarction and the lives lost due to this condition.[1–3] Unfortunately, however, LVSD and HF remain relatively common among survivors of myocardial infarction.[4] A Norwegian nation-wide cohort study between 2001-2009 in 63 583 patients with a first presentation of MI reported that 12.6% of patients were hospitalised or died because of HF over a median follow-up of 3.2 years.[5] This is because a large proportion of patients are not eligible for reperfusion therapy (i.e. those with non ST-segment elevation myocardial infarction) and many patients suffer recurrent episodes of infarction, leading to cumulative left ventricular damage over time.

Prior to reperfusion therapy, the major therapeutic breakthrough in myocardial infarction was the demonstration that angiotensin converting enzyme (ACE) inhibitors, given to prevent adverse “remodelling” (progressive dilatation and decline in systolic function) in high risk patients, reduced the likelihood of developing heart failure and the risk of death.[6] These benefits were seen in three seminal trials - Survival And Ventricular Enlargement (SAVE, with captopril), Acute Infarction Ramipril Efficacy (AIRE) and TRAndolapril Cardiac Evaluation Study (TRACE).[6–8] In the Valsartan in Acute Myocardial Infarction Trial (VALIANT), the angiotensin receptor blocker (ARB) valsartan was shown to be as effective as captopril used at the same dose as in SAVE.[9] ACE inhibitors and ARB work by interrupting one of the neurohumoral systems activated in patients who have sustained ventricular injury after infarction (the renin-angiotensin-aldosterone system or RAAS) and their benefits in high-risk myocardial infarction patients parallel similar benefits in patients with chronic heart failure due to left ventricular systolic dysfunction or reduced ejection fraction (HF-REF).[10–12] Indeed, four different neurohumoral antagonists (ACE inhibitors or angiotensin receptor blockers, beta-blockers and mineralocorticoid receptor antagonists) are life-saving in both conditions.[6–19] This is perhaps not surprising in that one of the commonest reasons patients develop chronic HF-REF is because of myocardial infarction weeks, months, years or decades previously. Many patients progress through an asymptomatic phase of progressive left ventricular remodelling and neurohumoral activation after myocardial infarction before developing symptomatic HF-REF. Another seminal trial showed that identification and treatment of patients with asymptomatic LVSD with an ACE inhibitor may delay or prevent the development of symptomatic HF-REF.[20] That trial was the prevention arm of the Studies of Left Ventricular Dysfunction (SOLVD-P), the sister study of SOLVD-T which demonstrated the benefit of the ACE inhibitor enalapril in a broad spectrum of patients with symptomatic HF-REF. As has been said (using old-fashioned terminology), there is a continuum “from the coronary care unit (CCU) to congestive cardiac failure (CCF)” and ACE inhibitors are beneficial across this continuum. The earliest indication of the potential benefit of ACE inhibitors or ARB in high-risk survivors of myocardial infarction was their ability to attenuate adverse left ventricular remodelling.[21,22]

It is important to recognise that not all of the neurohumoral systems which are activated in patients after myocardial infarction (and in heart failure) are harmful and some endogenous neurohumoral systems may be protective. The best recognised of these are the natriuretic peptides. A- and B-type natriuretic peptides are secreted by the heart when it is stressed and these peptides promote vasodilation (reducing left ventricular wall stress), stimulate renal sodium and water excretion (i.e. antagonising the retention of salt and water characterising heart failure) and inhibit pathological growth i.e. hypertrophy and fibrosis (key components of the adverse left ventricular remodelling that occurs after infarction and in heart failure).[23] The augmentation of plasma levels of endogenous natriuretic peptides can be achieved through inhibition of neutral endopeptidase, also known as neprilysin (NEP), which is the enzyme responsible for the breakdown of natriuretic peptides. Recently, in PARADIGM-HF, the addition of neprilysin inhibition to blockade of the RAAS (using sacubitril/valsartan – formerly known as LCZ696), compared with RAAS blockade alone (using the ACE inhibitor enalapril), reduced the risk of heart failure hospitalisation and death in patients with HF-REF.[24] These exciting findings may lead to a new approach to the treatment of heart failure, with an angiotensin receptor neprilysin inhibitor (ARNI) replacing an ACE inhibitor or ARB as one of the fundamental treatments for this condition.[25] We believe that the same approach may be beneficial in high-risk survivors of myocardial infarction. Recently, sacubitril/valsartan was shown to ameliorate adverse left ventricular remodelling in an experimental model of acute myocardial infarction.[26]

The objective of the present proposal is to obtain information, which is currently not available, on the cardiac effects of sacubitril/valsartan in patients with LVSD, better characterise the neurohumoral actions of sacubitril/valsartan and gather “proof-of-concept”, mechanistic, evidence in humans to support adoption of this new treatment in patients at high risk after myocardial infarction as a result of residual LVSD. Surprisingly, there is currently limited evidence about how sacubitril/valsartan works in humans. PARADIGM-HF was a large pragmatic mortality/morbidity trial with no mechanistic sub-studies and this is also true of a ongoing trial (PARADISE-MI) in acute myocardial infarction. Moreover, both trials either used or will use an ACE inhibitor (enalapril and ramipril, respectively), rather than an ARB as the active comparator for sacubitril/valsartan; use of valsartan in our study will allow us to precisely define the effects of neprilysin inhibition. A-type (or atrial) natriuretic peptide (ANP), C-type natriuretic peptide (CNP) and adrenomedullin are substrates for neprilysin and may play a role in the action of sacubitril/valsartan but have not been measured in existing clinical trials (in part because of the instability of these peptides and unfeasibility of measuring them in multi-centre, multi-national trials). Indeed, ANP and CNP are more specific substrates for neprilysin than BNP. As has been mentioned above, cardiac fibrosis appears to be important in the process of LV remodelling in patients with asymptomatic LVSD and the development of HF-REF and is reflected in circulating biomarkers which may be influenced by sacubitril/valsartan.

1.2 Study Rationale – hypothesis

We hypothesise that administration of sacubitril/valsartan, a first in class ARNI, to patients with LVSD (LVEF ≤40%) post myocardial infarction and no evidence of heart failure will result in a greater reduction in the degree of adverse remodelling, than the current standard of care, ARBs.

2 Study Objectives

2.1 Primary objective

To investigate the potential benefit of sacubitril/valsartan compared to the current standard of care, valsartan, in attenuating adverse left ventricular remodelling in high risk asymptomatic patients post myocardial infarction as a result of residual left ventricular systolic dysfunction (i.e. a reduced LVEF without symptoms or signs of heart failure).

2.2 Secondary objectives

To provide understanding of the cardiac effects and mechanisms of action of sacubitril/valsartan in patients with LVSD. This will help understanding of the clinical benefits seen in PARADIGM-HF.

3 Endpoints

3.1 Primary Endpoints

The primary endpoint is the change in indexed left ventricular end-systolic volume (LVESVI), from baseline to 12 (- 3 months +6 months)months, based on cardiac magnetic resonance imaging measurements.

3.2 Secondary Endpoints

- Change in other cardiac magnetic resonance imaging-based metrics (indexed LV end-diastolic volume, LVEF, LV mass) of LV remodelling from baseline to 12 (- 3 months +6 months) months.
- Changes in hsTnI from baseline to 12 (- 3 months +6 months) months.
- Changes in NT-proBNP from baseline to 12 (- 3 months +6 months) months.
- Change in patient well-being as assessed by a patient global assessment questionnaire.

3.3 Tertiary Endpoints

- Changes in biomarkers of LV remodelling (sST2, Galectin 3, TIMP-1, MMP-9, Type III Procollagen Peptide and GDF-15) from baseline to 12 (- 3 months +6 months) months.
- Changes in neurohormonal levels (BNP, MR-proANP, C-terminal ANP, CNP, MR-proADM, cGMP, endothelin-1, neprilysin antigen, renin and aldosterone) from baseline to 12 (- 3 months +6 months) months.

4 Investigational Plan

4.1 Study Design

This is a single-centre prospective, randomised, double-blind, active-comparator trial designed to evaluate the effect of sacubitril/valsartan compared to valsartan on LV remodelling post myocardial infarction. We will study patients after myocardial infarction (both ST elevation and non-ST elevation) who receive the usual standard of care (including beta-blockers and mineralocorticoid receptor antagonists) who are at high risk of developing symptomatic HF-REF. Potential study participants will have suffered an acute myocardial infarction at least 3 months prior to recruitment, have a LVEF ≤ 40% as measured by transthoracic echocardiography without any symptoms of HF (NYHA≥2), be tolerant of a minimum dose of ACE inhibitor/ARB (ramipril 2.5mg BD or equivalent) and be treated with a beta-blocker unless not tolerated or contraindicated. Study participants must also be haemodynamically stable, defined as systolic blood pressure (SBP) ≥100mmHg.

We will recruit patients with asymptomatic LVSD identified at least 3 months after myocardial infarction to ensure resolution of "stunning" (reversible LVSD). This will distinguish our study from the PARADISE-MI trial which will enrol patients up to 7 days after acute infarction and does not require all patients to have LVSD.[27] We did not consider it ethical to carry out a trial like the one proposed in patients with HF-REF as sacubitril/valsartan has already been shown to be definitively superior to RAAS blockade alone in those patients.[24]

After assessing eligibility during screening, consenting patients who meet the study inclusion and exclusion criteria will be randomised in a double blinded 1:1 fashion to sacubitril/valsartan (target dose 97mg/103mg BD) or valsartan (target dose 160mg BD) and matching placebo. All patients will require to be tolerant (determined by clinical condition, SBP, and renal function) of a minimum dose of ACE inhibitor/ARB (ramipril 2.5mg BD or equivalent –*Table 4-1*) prior to randomisation. If on a lower dose at time of first screening, patients may be rescreened after an observed period of up-titration to a dose of ramipril 2.5mg BD or equivalent in-line with current standard of care and local practice. In order to minimise the potential risk of angioedema due to overlapping ACE and NEP inhibition all patients prescribed an ACE inhibitor pre-randomisation will undergo a minimum 36-hour "washout" period following randomisation prior to first dose of study drug.

Table 4-1 Total daily doses of commonly used ACE inhibitors or ARBs corresponding to ramipril 2.5mg BD (dose level 2 of study drug)

ACE inhibitor	Dose	ARB	Dose
Captopril	100mg	Candesartan	16mg
Enalapril	10mg	Irbesartan	150mg
Lisinopril	10mg	Losartan	50mg
Perindopril	4mg	Valsartan	160mg
Trandolapril	2mg		

Three dose levels of study medication will be administered in a stepwise titration (*Table 4-2*). Study drug will be started at dose level 2 (sacubitril/valsartan 49mg/51mg BD or valsartan 80mg BD) and up-titrated after 4 weeks to dose level 3 (sacubitril/valsartan 97mg/103mg BD or valsartan 160mg BD) if tolerated as assessed by clinical review (SBP and symptomatic hypotension) and laboratory evaluation (renal function). Patients already on a higher dose equivalent of ACE-inhibitor/ARB at dose level 2 (*Table 4-1*), may start at dose level 3 at investigators discretion taking into consideration the patient's prior dose level of ACE-inhibitor/ARB and clinical condition (SBP and renal function).

A dose level 1 (sacubitril/valsartan 24mg/26mg B.D) will be available during the double-blind period of the trial should patients require down-titration but the aim is to maintain the patient on the target dose level 3 for as long a duration as possible during the trial. (*Figure 4-1 Study drug initiation and up-titration*) This dose can be also be considered at the time of randomisation with a two-step titration to target dose over visits 3 and 4 if systolic BP at visit 1 is ≥100 to 110mmHg. Additionally, dose level 1 should be considered as initial dose in patients with moderate renal impairment (eGFR 30-60 ml/min/1.73 m^2).

Table 4-2 Study drug dose levels during treatment period

Dose Level	Sacubitril/Valsartan arm	Valsartan arm
1	24mg/26mg B.D.	40mg B.D.
2	49mg/51mg B.D.	80mg B.D.
3	97mg/103mg B.D.	160mg B.D.

Figure 4-1 Study drug initiation and up-titration

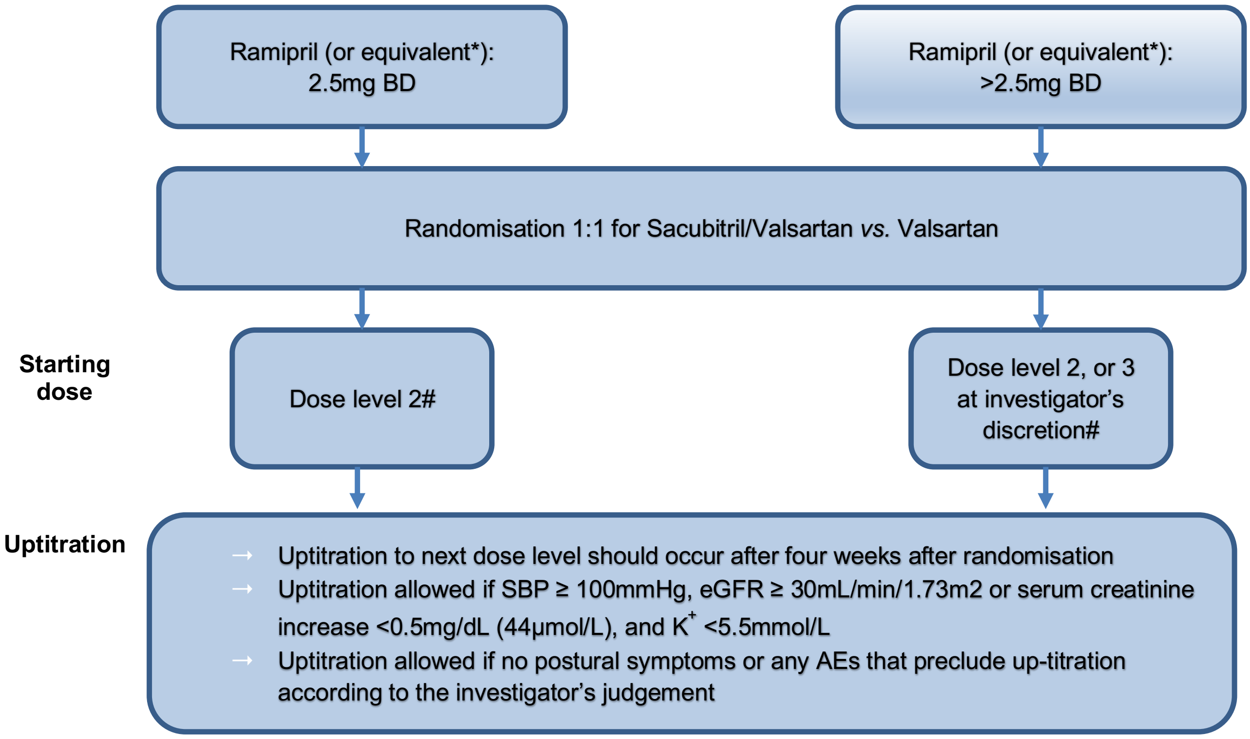

** Equivalent doses detailed in Table 4-1*
# Dose level 1 can be considered for patients with SBP ≥100 to 110mmHg and/or moderate renal impairment (eGFR 30-60 ml/min/1.73 m^2) at time of randomisation
+ down titration from dose level 3 to dose level 2 permitted during extension of treatment times in response to COVID-19 pandemic

Following randomisation the aim should be to up-titrate patients to target study drug dose level 3 (*Table 4-2*) after 4 weeks (Visit 4). Slower up-titration will be permitted if necessary to ensure patient safety and tolerability. Patients will be allowed to stay at dose level 1 or 2 as maintenance dose however efforts should be made to maintain patients on the target dose level 3 or maximally tolerated dose level for as long a duration as possible during the trial. Adjustments to study drug dose level should be based on safety and tolerability with a focus on a) symptomatic hypotension, b) clinically significant decline in renal function and c) hyperkalaemia (Table 4.3).

The decision to up-titrate to the next dose level at any study visit will be made based on investigator discretion and may be made on the basis of results of the laboratory investigations (urea and electrolytes) from a previous visit or those performed in routine clinical care provided these are within 14 days of the study visit. Participants will have laboratory investigations (urea and electrolytes) performed at each study visit but may be advised to await communication from the investigator prior to taking their next study drug dose. It is anticipated that results will be available within several hours of the study visit occurring and that study participants will be contacted within a maximum of 12 hours from the time of the study visit. The investigator will make all reasonable attempts to contact participants within this time period. Based on previous results and investigator discretion, the decision to up-titrate may be delayed until the results of laboratory investigations are available in which case participants will continue on current study dose until the results are available.

Treatment guidance for management of nephrotoxicity, hyperkalaemia and hypotension are provided in section 6.8.1, 6.8.2 and 6.8.3 respectively.

Table 4-3 Safety monitoring criteria that must be met for dose up-titration

**Parameter**	**Criteria**
Blood pressure	SBP ≥ 100mmHg
Renal function	eGFR ≥ 30mL/min/1.73m^2 or serum creatinine increase <0.5mg/dL (44μmol/L)
Serum potassium	K^+ <5.5mmol/L
AEs or symptoms	No postural symptoms or any AEs that preclude up-titration according to the investigator's judgement

Study patients will remain on the allocated study drug for a period of 12 (- 3months or + 6months) months after which their involvement in the trial will end. In response to the covid-19 pandemic down titration from dose level 3 to dose level 2 is permitted. Following their involvement in the trial they will be converted to an ACE-inhibitor or ARB as per local guidelines at an equivalent dose to their final study drug dose level. It is anticipated that recruitment of 100 patients will take 18 months with the final patient visit occurring 12 months following this meaning a total trial duration of 30 months.

4.2 Rationale of key elements of the study design

Current guidelines advocate the use of ACE-inhibitors (or angiotensin receptor blockers if ACE-inhibitor contraindicated/not tolerated) along with beta-blockers and

mineralocorticoid receptor antagonists to reduce morbidity and mortality after myocardial infarction.[25] The role of ACE-inhibitors/ARBs in preventing adverse remodelling after myocardial infarction is well established. A meta-analysis of 16 trials examining ACE-inhibition after myocardial infarction demonstrated a significant improvement in reduction of left ventricular volumes at both short and long-term follow up in patients with reduced left ventricular ejection who were administered an ACE-inhibitor.[28] Similarly, in patients with asymptomatic LVSD, usually as a result of remote myocardial infarction, ACE inhibitor therapy attenuates the progressive left ventricular remodelling.[29]

To date, the administration of an ARNI has not been studied in patients after myocardial infarction. Sacubitril/valsartan (brand name "Entresto") is a first-in-class ARNI, which has been studied in a pivotal trial in the setting of chronic HF-REF in patients with LV ejection fractions of ≤40%.[24] Sacubitril/valsartan (at a target dose of 97mg/103mg BD) when compared to the gold standard ACE-inhibitor, enalapril (target dose 10mg BD), was associated with a significant reduction in cardiovascular death and hospitalisation for heart failure. Recently, sacubitril/valsartan was shown to have favourable effects on left ventricular remodelling in an experimental rat model of acute myocardial infarction, although in this study sacubitril/valsartan was compared to control rather than an ARB.[26]

The target dose (97mg/103mg BD) of sacubitril/valsartan is based on the clinical benefit and safety results seen with this dose in PARADIGM-HF.[24] In this trial there was a high prevalence of ischaemic cardiomyopathy (60%), with 43% of patients having had a prior MI. This target dose delivers equivalent valsartan exposure (assessed by AUC) as valsartan 160mg BD and biomarker analysis (increase in ANP and cGMP) indicates that this dose delivers approximately 90% of its maximal NEP inhibition.[30] The target dose of valsartan (160mg BD) was shown to be as effective as the ACE inhibitor captopril (target dose 50mg three times daily) in the VALIANT trial at reducing mortality, recurrent myocardial infarction and hospitalisation for heart failure in patients with LVSD and/or HF post MI.[9]

CMR is the gold standard means of assessment of LV mass, volumes and ejection fraction. It has the additional benefit of allowing assessment of myocardial viability, myocardial fibrosis and regional dysfunction.[31] LVESVI has been shown to be a major determinant of survival after myocardial infarction.[32,33] The degree of LV remodelling and effect of treatment will be measured by the primary endpoint of the change in LVESVI from baseline to 12 (- 3months or + 6months) months.

5 Patient Population

This study will recruit male and female patients aged ≥18 years with asymptomatic left ventricular systolic dysfunction (i.e. no evidence of clinical and/or radiological heart failure) > 3 months post acute myocardial infarction.

Patients will be required to satisfy the following criteria. Eligibility waivers to the inclusion / exclusion criteria are NOT permitted.

5.1 Inclusion Criteria

- Acute myocardial infarction at least 3 months prior to recruitment
- LVEF ≤ 40% as measured by transthoracic echocardiography
- Ability to provide written, informed consent
- Age ≥18 years
- Tolerance of a minimum dose of ACE inhibitor/ARB (ramipril 2.5mg BD or equivalent)
- Treatment with a beta-blocker unless not tolerated or contraindicated

5.2 Exclusion Criteria

- Contraindication to CMR (ferrous prosthesis, implantable cardiac device or severe claustrophobia)
- Clinical and/or radiological heart failure (NYHA≥2)
- Symptomatic hypotension and/or systolic blood pressure <100mmHg
- eGFR < 30 mL/min/1.73m^2 and/or serum potassium >5.2mmol/L
- Persistent/permanent atrial fibrillation
- History of AMI within last 3 months
- History of hypersensitivity or allergy to ACE-inhibitors/ARB
- History of angioedema
- Known hypersensitivity to the active study drug substances, contrast media or any of the excipients
- Obesity (where body girth exceeds MRI scanner diameter)
- Pregnancy, planning pregnancy, or breast feeding
- Inability to give informed consent or comply with study protocol
- Evidence of hepatic disease as determined by any one of the following: AST or ALT values exceeding 2 x ULN at Visit 1, history of hepatic encephalopathy, history of oesophageal varices, or history of portacaval shunt
- History of biliary cirrhosis and cholestasis
- Active treatment with cholestyramine or colestipol resins
- Active treatment with lithium or direct renin inhibitor
- Participation in another intervention study involving a drug or device within the past 90 days (co-enrolment in observational studies is permitted)

5.3 Women of child bearing potential

Women of childbearing potential must be currently adhering to, or be willing to use, highly effective birth control methods for study treatment duration and for seven days thereafter including:

- Combined hormonal contraception (oestrogen and progestogen containing medication) either orally, intravaginally, or transdermally
- Progesterone only hormonal contraception either orally, injected, or implanted
- Progesterone only hormonal contraception either orally, injected, or implanted
- Intrauterine device (IUD)
- Intrauterine hormone release system (IUS)
- Bilateral fallopian tube occlusion

- Vasectomised partner
- Complete sexual abstinence where this is their preferred and usual lifestyle

Women of child bearing potential (WOCBP) comprises women who have experienced menarche and who have not undergone successful surgical sterilisation (hysterectomy, bilateral tubal ligation, or bilateral oophorectomy) or who are not post-menopausal defined as:

- Women who have had amenorrhea for ≥ 12 consecutive months (without another medical cause) and who have a documented serum follicle-stimulating hormone (FSH) level > 35 mIU/mL.
- Women who have irregular menstrual periods and a documented serum FSH level > 35 mIU/mL.
- Females of childbearing potential must have a negative pregnancy test within 7 days prior to the initiation of treatment.

Pregnancy tests will be performed in WOBCP at the following visits: Visit 1 (week 0), 4 (week 4), 6 (week 14), 7 (week 26), 8 (Week 39), and 9 (week 52). Should this test be positive, then the patient will be immediately withdrawn from the trial and study drug will be discontinued.

5.4 Males whose partner could become pregnant

To be eligible for this study participants will be taking an ACEI or ARB. These are similar to the study medicines and with the information currently available there is no indication that men should avoid fathering a child whilst taking the study medicines. Males whose partner may have become pregnant during the study must be advised to inform the investigator immediately. If the partner consents data may be collected about the pregnancy and the health of the partner and their baby.

5.5 Pregnancy

The Investigator must ensure that all patients are aware at the start of a clinical trial of the importance of reporting all pregnancies (in themselves and their partners) that occur whilst being treated with IMP and occurring up to 1 month after the last IMP administration. The Investigator should offer counselling to the patient and/or the partner, and discuss the risks of continuing with the pregnancy and the possible effects on the foetus. Monitoring of the patient and the baby should continue until the conclusion of the pregnancy, if the patient or patient's partner has consented to this. See section 10.7 for information on pharmacovigilance reporting requirements.

6 Study Treatment

6.1 Investigational and control drugs

All eligible patients will be randomised 1:1 to either sacubitril/valsartan titrated to a target dose of 97mg/103mg B.D. or valsartan titrated to a target dose of 160mg BD

in a double blinded manner for the duration of the study. Patients will continue on optimal standard of care background therapy to treat their index MI and any co-morbid conditions in accordance with local guidelines with the exception of an ACE inhibitor or ARB as this will be replaced by study drug. In order to minimise the potential risk of angioedema due to overlapping ACE and NEP inhibition all patients prescribed an ACE inhibitor pre-randomisation will undergo a 36-hour “washout” period following randomisation prior to first dose of study drug. The use of open label ACE inhibitor or ARB in addition to the randomised study drug is strictly prohibited for the duration of the trial.

In order for adequate blinding of treatment allocation, patients will be required to take a total of two tablets (one tablet from the sacubitril/valsartan or sacubitril/valsartan matching placebo pack and one tablet from the valsartan or valsartan matching placebo pack) twice a day for the duration of the study.

The following study drugs will be provided:

- Sacubitril/valsartan 24mg/26mg, 49mg/51mg and 97mg/103mg tablets and matching placebo.
- Valsartan 40mg, 80mg and 160mg tablets and matching placebo.

Patients will be supplied with sufficient medication to last to the next visit including a small overage at visits 1, 4, 6 and 7. At each study visit where medicines are dispensed, patients will be asked to return any unused study drug and this will be returned to the pharmacy. In response to the COVID-19 pandemic re supply in person at the CRF or through home delivery at unscheduled intervals until the final MRI is performed (up to a maximum of 18 months or end of study).

Patients will be advised to take their morning study drug doses at approximately 08:00 (8 AM) and their evening dose 12 hours later at approximately 20:00 (8PM). The study medicines should be swallowed whole with water and can be taken with or without food. If a patient misses a dose of either study drug they should skip the missed dose and take the next one at the scheduled time.

Compliance will be promoted through use of tablet counts and investigators must emphasise the importance of compliance for both patient safety and study validity. If, for any reason, patients are unable to take study drug as prescribed then they will be told to contact the investigators for further advice.

6.2 Study Drug storage and supply

Investigational medicinal product (IMP) supplies will only be released to study sites by the sponsor once all the appropriate regulatory and governance approvals are in place.

All study medicines must be stored in a locked, secure area with access limited to the Investigator and authorised site staff. Study supplies should be used as directed in the study protocol and not be supplied to any persons other than study participants. Study medicines must be stored at room temperature.

6.3 Drug accountability requirements

The Investigator or designee must maintain accurate records of all study IMP movements for accountability purposes. They should include dates, quantities, batch numbers and expiry. Records must document adequately that:

- the patients were provided the doses specified by the protocol/amendment(s)
- all study drug provided was fully reconciled.

Unused study drug must not be discarded or used for any purpose other than the present study. Further information on storage requirements and supply arrangements is provided in the study specific IMP Management and Accountability Manual.

6.4 Treatment arms

Patients will be assigned to one of the following two treatment arms in a ratio of 1:1 at study visit 1:

- Sacubitril/valsartan at dose levels 1-3 (24mg/26mg, 49mg/51mg and 97mg/103mg BD)
- Valsartan at dose levels 1-3 (40mg, 80mg and 160mg BD)

6.5 Permitted dose adjustments and discontinuation of study treatment

Every attempt should be made to maintain patients at the target study drug dose level throughout the trial. If the patient does not tolerate the target study drug dose level the investigator can, if appropriate, adjust concomitant background medications for co-morbid conditions to rectify the situation, and if necessary down titrate to the next lower study drug dose level. Any dose-adjustment of disease-modifying background therapy, e.g. beta-blockers or MRAs is discouraged.

Down-titration of the study drug at any time will be allowed based on the safety and tolerability criteria defined in Table 4-3 and section 6.8. If down-titration is necessary, the patient should be down-titrated to the next lower dose level (Table 4-2) The patient may continue receiving the lower dose level for a recommended period of 1 to 4 weeks before re-challenging the patient with the next higher dose level. For example, a patient who encounters tolerability problems at the target dose level (dose level 3), should receive the study drug at dose level 2 for 1 to 4 weeks. Then, he/she should be re-challenged with up-titration back to dose level 3. If the tolerability issues are not alleviated despite down-titration by one dose level, the investigator may lower the study drug dose further to the next lower level for 1 to 4 weeks, up to temporary withdrawal of the study drug. Study drug should be reintroduced in those who temporarily discontinue it as soon as medically justified in the opinion of the investigator. Again, once stable, the patient should be re-challenged with up-titration to the next higher dose level every 1 to 4 weeks in an attempt to bring back the patient gradually to the target study drug dose level (dose level 3). The investigator may choose the next dose level for down- or up-titration according to his or her judgement.

In some instances, according to the safety and tolerability criteria and the investigator's judgment, dose level 1 or 2 could be maintained if he/she considers that the patient's condition would not allow any further up-titration to the target dose of study medication (level 3). In this case it would be acceptable to maintain the patient at dose level 1 or level 2, whichever is the higher and tolerated dose level by the patient.

Any dose change must be recorded on the dosage administration record on the eCRF.

Should a patient develop symptomatic heart failure during the study they will be offered open-label sacubitril/valsartan. Patients starting open-label sacubitril/valsartan or withdrawing from study medication (or study follow-up) ≥6 months after randomisation will be asked to undergo an end-of-study CMR examination (patient withdrawing before 6 months will not be asked to have a second CMR as an effect of LV remodelling is unlikely to be detected before this time point).

The emergence of the following circumstances will require permanent study drug discontinuation:

- Withdrawal of informed consent
- Investigator thinks that continuation of study drug would be detrimental to the patient's well-being
- Suspected or confirmed occurrence of clinically significant angioedema. A patient with any signs or symptoms of clinically significant angioedema should be thoroughly evaluated by the investigator
- Pregnancy and post-pregnancy during lactation period

The emergence of the following circumstances will require temporary or permanent discontinuation (study drug may be restarted once these circumstances no longer exist):

- Use of an open label ACE inhibitor, ARB, direct renin inhibitor, lithium, cholestyramine or colestipol resins (*Table 6-1*)
- Any laboratory abnormalities that in the judgment of the investigator warrant discontinuation of study drug after taking into consideration the patient's overall condition

Study drug may be discontinued at the investigator's discretion if any of the following occurs:

- Any severe suspected drug-related AE
- Any other protocol deviation that results in a significant risk to the patient's safety
- Subsequent clinical need for implantable cardiac device insertion (e.g. permanent pacemaker, implantable cardioverter defibrillator, cardiac resynchronisation therapy)

6.6 Concomitant medications

Prior to randomisation and at each study visit a list of the patient's current medications will be reviewed and the patient will be instructed to inform the investigator about any new medications he/she takes after enrolment in the study. Each new medication must be individually assessed against the exclusion criteria and list of prohibited medication below (*Table 6-1*)

Medications known to raise potassium levels

Potassium-sparing diuretics, potassium supplements and any other medications known to raise potassium levels should be used with caution while the patient is receiving the study drug due to the increased possibility of occurrence of hyperkalemia. Patient's potassium levels will be monitored regularly, especially in those who are receiving these medications.

Phosphodiesterase-5 (PDE-5) inhibitors

PDE-5 inhibitors should be used with caution while the patient is receiving study medication due to the increased possibility of the occurrence of hypotension.

HMG-CoA reductase inhibitors (statins)

Caution is recommended when co-administering sacubitril/valsartan with atorvastatin or other statins because of the potential to raise its plasma level.

Non-steroidal anti-inflammatory drugs (NSAIDs)

In elderly patients, volume-depleted patients (including those on diuretic therapy), or patients with compromised renal function, concomitant use of sacubitril/valsartan or valsartan and NSAIDs may lead to an increased risk of worsening of renal function. Close monitoring of renal function as per study protocol will take place in all patients.

OATP and MRP2 transporters

The active metabolite of sacubitril (LBQ657) and valsartan are OATP1B1, OATP1B3, OAT1 and OAT3 substrates; valsartan is also a MRP2 substrate. Therefore, co-administration of Entresto with inhibitors of OATP1B1, OATP1B3, OAT3 (e.g. rifampicin, ciclosporin), OAT1 (e.g. tenofovir, cidofovir) or MRP2 (e.g. ritonavir) may increase the systemic exposure of LBQ657 or valsartan. Appropriate care should be exercised when initiating or ending concomitant treatment with such medicinal products.

Prohibited medication

Use of the treatments displayed in *Table 6-1* is NOT allowed after the start of study drug due to safety reasons, unless the actions specified are taken.

Table 6-1 Prohibited medications

Medication	Action taken

Any ACE inhibitor	Discontinue study drug. The open label ACE inhibitor must be stopped for ≥36 hours prior to re-initiation of study drug
Any ARB	Discontinue study drug. The open label ARB must be stopped prior to re-initiation of study drug
Any direct renin inhibitor	Discontinue study drug. The open label direct renin inhibitor must be stopped prior to re-initiation of study drug
Entresto (commercially available sacubitril/valsartan)	Discontinue study drug. The open label Entresto must be stopped for ≥36 hours prior to re-initiation of study drug
Lithium	Discontinue study drug. Lithium must be stopped prior to re-initiation of study drug
Cholestyramine or colestipol resins	Discontinue study drug. Cholestyramine or colestipol resins must be stopped prior to re-initiation of study drug

The concomitant use of open-label ACE inhibitor, ARBs, commercially available sacubitril/valsartan (Entresto) or a direct renin inhibitor is **strictly prohibited** while the patient is receiving study drug. If the addition of an ACE inhibitor, ARB, Entresto or direct renin inhibitor is necessary, then study drug must be temporarily discontinued. If the patient is to be started on open-label ACE inhibitor or Entresto, the study drug must be stopped ≥36 hours prior to initiating ACE inhibitor or Entresto. If study drug is to be re-started, the open-label ACE inhibitor or Entresto must also be stopped ≥ 36 hours prior to re-initiating study drug. ARBs or a direct renin inhibitor should be stopped prior to resuming study drug.

6.7 Identification of participants and treatment assignment

Potentially eligible patients will be identified through the “post MI LVSD” or general cardiology out-patient clinics throughout NHS Greater Glasgow and Clyde (NHS GG&C) and surrounding health boards. Participants will be identified from electronic searching of the cardiac patient databases by a clinical research fellow, who is a member of the direct clinical care team. They will also be identified by clinicians and nurses responsible for the routine care of these patients, for example cardiac rehabilitation nurses, cardiology pharmacists and cardiologists.

Patients will be considered if they have suffered a myocardial infarction at least 3 months prior to randomisation.

The definition of myocardial infarction will be in line with the guidelines provided by the European Society of Cardiology and the third Universal definition of myocardial infarction.[34,35] Patients will require to meet the following criteria:

- Detection of rise and/or fall of cardiac biomarker values (preferably troponin) with at least one value above the 99th percentile of the upper reference limit and with at least one of the following:

- Symptoms of ischaemia;
- New or presumably new significant ST-T changes or new LBBB;
- Development of pathological Q waves in the ECG;
- Imaging evidence of new loss of viable myocardium, or new regional wall motion abnormality;
- Identification of an intracoronary thrombus by angiography or autopsy.

All patients without obvious contraindications to enrolment based on review of case-notes will be approached with regards to taking part in trial-screening. They will be provided with a patient information sheet detailing the trial and given the opportunity to ask questions prior to screening. Patients who are agreeable will be consented and will undergo screening transthoracic echocardiography (TTE), performed by one operator (KD), in order to estimate LVEF (Simpson's biplane rule). The TTE will be performed at least 3 months after the index myocardial infarction. Patients with insufficient endocardial definition to allow accurate planimetry will be excluded from recruitment. This screening process will continue until the pre-specified target population (n=100) has been recruited. Patients who initially fail screening may be re-screened once more. Re-screened patients will be allocated a new patient number. If the patient has failed screening and is re-screened within 3 months of initial screening date the initial screening echocardiogram measurement of ejection fraction will be permitted to used for purposes of eligibility unless felt clinically relevant to repeat scan.

A log of all patients screened for eligibility will be completed. Anonymised information will be collected including:

- Age
- Gender
- Ethnicity
- Whether the patient is recruited or not recruited to the study

This information will be collated in the study database. Screened patients who are not recruited either because they are ineligible or because they decline participation will also have the following information recorded:

- The reason not eligible for study participation OR
- Where eligible, reason declined

However, the right of the patient to refuse consent without giving reasons will be respected. This anonymised information will be stored in a locked filing cabinet in the BHF Glasgow Cardiovascular Research Centre (GCRC).

Assenting patients who meet the eligibility criteria and without any contraindications to taking part in the study will be given information regarding the trial as detailed in the patient information sheet. If agreeable to taking part in the trial, then written, informed consent for will be sought. Enrolled patients will be allocated a unique patient identifier number which will last for the duration of the entire trial. The assessment of eligibility and the informed consent process will be undertaken by a member of the research team who is qualified by training and experience in taking informed consent to good clinical practice (GCP) standards. Informed, written consent is necessary prior to randomisation. A copy of the consent form will be given

to the patient and another will be uploaded to the patient's online case notes. The original consent form will be filed in the study file and sites will be required to scan and upload the consent forms into a secure study database for each consented patient.

After all baseline data has been collected and the patient has been confirmed as eligible for randomisation, the patient will be randomised via the study web portal. Randomisation will be stratified on the basis of LVESVI ≤45ml/m^2 / >45ml/m^2 as measured by CMR, and whether or not the patient is being prescribed diuretics at baseline. The portal will inform the researcher of the treatment packs to be allocated to the patient. Randomisation may occur up to three months after screening echocardiography.

6.8 Monitoring of potential side effects

6.8.1 Management of nephrotoxicity

Both study drugs are potentially nephrotoxic and close monitoring of renal function (serum urea, creatinine and electrolytes) for the duration of the trial will be mandatory. This will include baseline measurements along with measurements prior to and after the introduction of the study drug and after any dose change. Measurements will also be taken at regular intervals for the duration of the trial. Alert ranges for laboratory and other test abnormalities are included in Appendix 1. Additional measurements will be used if clinically indicated. All results will be reviewed in a timely manner when available and if action is required this will be expedited immediately. Each patient will have their renal function (along liver function tests) checked at the following time-points:

- Visit 1: Week 0 (pre-randomisation)
- Visit 2: Week 1 (± 3 days)
- Visit 3: Week 2 (± 3 days)
- Visit 4: Week 4 (± 3 days - pre up-titration of study drug)
- Visit 5: Week 5 (± 3 days - 1 week post up-titration of study drug)
- Visit 6: Week 14 (± 7 days)
- Visit 7: Week 26 (± 7 days)
- Visit 8: Week 39 (± 7 days)
- Visit 9: Week 52 (- 3 months + 6 months in reponse to COVID-19)

If, at any time after randomisation, eGFR decreases by ≥ 25% from baseline (or if serum creatinine concentration increases to 221 µmol/L), potentially reversible cases of renal dysfunction will be sought including: non-steroidal anti-inflammatory drug intake, antibiotics, or other nephrotoxic medications; hypovolaemia; and urinary tract infection. If felt to be appropriate, the study drug dose can be reduced and continued with regular monitoring of renal function. If a patient's eGFR decreases by ≥ 40% from baseline (or if serum creatinine concentration rises above 265 µmol/L, then this is an indication to stop the study drug. Thereafter, serum creatinine assessments will have to be repeated at least weekly until levels return to acceptable values. Re-challenging with the study drug at a lower dose level will be considered if deemed clinically safe to do so.

6.8.2 Management of hyperkalaemia

Patients with elevated potassium value will be managed according to the corrective actions outlined below.

Serum potassium > 5.3 and less than or equal to 5.5 mmol/L

- Confirm potassium concentration in a non-haemolysed sample
- Reinforce low potassium diet and restriction of food/drinks with high potassium content
- (e.g. orange juice, melon, bananas, low-salt substitutes etc.)
- Review medical regimen (including dietary supplements and over-the-counter medications) for agents known to cause hyperkalaemia. Consider reduction in dose or discontinuation of these agents:
    - Potassium-sparing diuretics (e.g. amiloride and triamterene) including in combination products with thiazide or loop diuretics
    - Potassium supplements, e.g., potassium chloride
    - Salt substitutes
    - Non-steroidal anti-inflammatory drugs (NSAIDs)
    - Cyclo-oxygenase-2 (COX-2) inhibitors
    - Trimethoprim and trimethoprim-containing combination products
    - Herbal Supplements: For example, Noni juice, alfalfa (Medicago sativa), dandelion (Taraxacum officinale), horsetail (Equisetum arvense), nettle (Urtica dioica), milkweed, lily of the valley, Siberian ginseng, hawthorn berries
- Repeat serum potassium measurement within 3 to 5 days
- If serum potassium remains > 5.3 and ≤ 5.5 mmol/L, regularly monitor serum potassium levels to ensure stability (suggested once monthly)
- Consider down-titration of study medication, according to investigator's medical judgment.

Serum potassium > 5.5 and < 6.0 mmol/L

- Confirm potassium concentration in a non-haemolysed sample
- Consider down-titration or temporarily discontinue study drug according to investigator medical judgment.
- Apply all measures outlined for serum potassium > 5.3 and ≤ 5.5 mmol/L
- Repeat serum potassium measurement after 2-3 days
- If serum potassium < 5.5 mmol/L, consider resumption of study drug at lower dose with repeat potassium within 5 days

*Serum potassium greater than or equal to 6.0 mmol/*L

- Immediately discontinue study drug
- Confirm potassium concentration in a non-haemolysed sample
- Urgently evaluate patient and treat hyperkalaemia as clinically indicated
- Apply all measures outlined for serum potassium > 5.3 and < 6.0 mmol/L
- Resumption of study drug will be decided on a case-by-case basis by the investigators.

6.8.3 Management of hypotension

Both study drugs can have the potential side effect of symptomatic hypotension and/or postural symptoms. Prior to any dose change blood pressure will be checked and up-titration will only occur if there is no symptomatic hypotension and systolic blood pressure is ≥ 100mmHg. If appropriate, at the investigators discretion adjustments can be made to doses of diuretic and/or concomitant antihypertensive agents to facilitate the patient’s ability to tolerate target study dose.

6.8.4 Management of angioedema

Angioedema is a potential side effect of combined RAAS and NEP inhibition. Angioedema has been reported in patients treated with sacubitril/valsartan. If angioedema occurs, study drug should be immediately discontinued and appropriate therapy and monitoring should be provided until complete and sustained resolution of signs and symptoms has occurred. It must not be re-administered. Any patients who experience angioedema during the trial will be withdrawn from the study.

6.9 Withdrawal of treatment

Any patient enrolled in the trial will be free to withdraw from the study at any time without giving reason and without prejudicing any further treatment/care. Following their involvement in the trial any patient withdrawing will be converted to an ACE-inhibitor or ARB as per local practice guidelines at an equivalent dose to their final study drug dose level.

6.10 Unblinding procedure

Breaking of the study blind in an emergency should only be performed where knowledge of the treatment is absolutely necessary for further management of the patient. In case of any acute illness or major surgery, the study medication should be discontinued. Emergency unblinding will be available at all times, via the telephone (Interactive Voice Response System, IVRS). Details are included in the study alert card. For each unblinding request an email alert will be sent to the appropriate members of the research team, including the Chief Investigator (CI).

Patients will be provided with an alert card at the randomisation visit after verbal consent is obtained. They will be asked to carry the alert card with them at all times and to show the card to any doctors or healthcare professionals who are involved in their care. Patients will also be asked at study visit 4 if they still have this and be provided with another if required. The Patient Alert Card will be collected from patients at the end of their involvement in the study.

Unblinding the treatment allocation may be required when reporting suspected unexpected serious adverse reactions (SUSARs) to the regulatory authorities. This will be performed by the Sponsor PV Office.

7 Assessment and management of risk

The risk to patients participating in the study will be minimised by compliance with the inclusion/exclusion criteria and close clinical monitoring.

Patients will be instructed not to take any RAAS blockade medications (ACE inhibitor or ARB) from the day they start study drug to avoid excess RAAS blockade. The importance of a minimum 36-hour washout period prior to commencement of study drug in patients receiving ACE-inhibitors prior to randomisation will be reiterated to minimise the risk of angioedema. The risk of discontinuation of pre-randomisation ACE inhibitors or ARBs will be minimal as the study treatment will be reflective of the typical dosing schedule of most ACE inhibitors and ARBs. All patients will be required to continue receiving the rest of their standard of care background cardiovascular medications.

In women of child-bearing potential, a possible risk of developmental toxicity cannot be excluded. Women of child-bearing potential should therefore use a highly effective method of contraception during study participation. If there is any question that the patient will not reliably comply, they should not be entered in the study. Pregnancy testing will be performed prior to randomisation and at regular intervals during the study in women of child-bearing potential.

Update March 2020 in response to the COVID-19 pandemic

The risk to patients of continuing study drug for longer than 12 months is felt to be very low. After 12 months involvement in the trial, patients will have been taking their allocated dose of study drug for 11 months. No issues with tolerability have been identified in this time period. Given this and the widespread use of the two approved treatments used in the trial, and their known safety profile, we believe that it is very unlikely that there will be any change in tolerability with longer exposure. Sacubitril/valsartan is currently licensed for long-term use in patients with symptomatic heart failure and reduced fraction, and valsartan is licensed for use in patients with left ventricular systolic dysfunction with, or without heart failure following myocardial infarction. Therefore, a short prolongation (up to 6 months) of the period of exposure to IMP is considered to be of low risk to the patient population enrolled in this trial; indeed each patient will be assessed for continued long-term treatment with one of these medications after the end of the trial.

Ongoing safety will be assessed by means of phone-calls to patients every 6-8 weeks.

Review of patient's electronic health care records will also be carried out at these timepoints to review any blood biochemistry carried out in primary or secondary care. Patients will be encouraged to contact the study team in the case of any change in health status during this time period.

To note – no WOCBP remain in the study for consideration of extension of IMP.

8 Visit schedule and assessments

Table 9-1 details all of the visits and indicates with an “x” when study assessments or procedures are performed. Patients should be seen for all visits at the designated time or as close to as possible. These visits windows are what investigators should endeavor to stick to and wherever possible should be met. Any visits out with these timeframes will not be considered protocol violations but will be dealt with in the statistical analysis plan.

8.1 Screening

Potentially eligible patients will be identified through the “post-MI LVSD” clinic or general cardiology out-patient clinics in NHS Greater Glasgow and Clyde (NHS GG&C) and surrounding health boards. Additionally, participants will be identified from electronic searching of the cardiac patient database by a clinical research fellow, who is a member of the direct clinical care team. They will also be identified by clinicians and nurses responsible for the routine care of these patients, for example cardiac rehabilitation nurses, cardiology pharmacists and cardiologists.

Potential participants will be approached in one of two ways:

(1) Approach in person by the clinical care team at a routine outpatient appointment. Those who demonstrate interest will be given the patient information sheet (PIS), and asked for verbal consent for their details to be passed to the research team. The PIS will contain a contact email address and telephone number to allow patients to “opt in” to the study or get in contact for further information. If there has been no contact from a patient 48 hours after being given the PIS the patient will be telephoned by a clinical fellow or research nurse to ascertain interest in participation in the study.

(2) Letter drop: letters posted to the patient by the principal investigator with information about the study. The same contact information will be provided for patients contacted by letter drop to allow them to opt in to the study. These patients will also be contacted 5-7 days after posting the letter by a clinical fellow to assess interest in participating in the study; Initial screening will be based on a telephone discussion with the potential patient who has expressed an interest in participating in the study supported by a review of their health records by a clinical fellow, as part of their routine clinical care team, and the research team. Where possible, any missing biochemical data required as an inclusion criterion will be ordered ahead of enrolment as part of standard of care if unrecorded in the last 6 months. This is to ensure appointments for screening echocardiography are only made for patients considered suitable to enter the study and avoid unnecessary inconvenience if they are not thought to be eligible based on routine information. Telephone discussions with the patient would also aim to ensure their ability to adhere with the trial, diagnostic tests, outcomes measures, and their ability to give informed consent. If the potential participant wishes to discuss any aspect of the study, they will be given the opportunity to do so with a member of the research team either over the phone or at the screening study visit and before consent is taken.

If agreeable and eligible on review of their medical case notes, assenting patients will then proceed to screening echocardiography. Patients will be consented for screening echocardiography and routine biochemical blood tests (U&Es and LFTs) if unrecorded in the last 6 months to ensure trial eligibility.

8.1.1 Echocardiography protocol

Transthoracic echocardiography (TTE) will be performed in the left lateral decubitus position. Images will be taken from the standard parasternal, apical and subcostal windows. A standard complete exam will be performed and images stored for off-line measurements.[36] LV ejection fraction will be calculated from apical 4 and 2 chamber views using Simpson's biplane rule. A cut-off of less than or equal to 40% will be used for inclusion in the trial. Contrast agents will not be used.

After screening echocardiography, suitable patients will be approached for consent to take part in the trial as described above. Patients who have consented will undergo the following tests at least 3 months post myocardial infarction and prior to randomisation to either sacubitril/valsartan or valsartan.

8.2 Pre-randomisation investigations

The following investigations will be performed prior to randomisation and all results recorded in the eCRF.

- Full physical examination – including measurement of height, weight and resting heart rate and blood pressure
- A record of the patients past medical history will be obtained. The presence of diabetes mellitus (Type-1 or Type-2), hypertension, hyperlipidaemia, cerebrovascular disease, peripheral vascular disease, chronic obstructive pulmonary disease and smoking history will be recorded. Prescribed medication pre-randomisation will also be recorded.
- 12-lead ECG
- Spot urine collection
- Venepuncture – 50ml of venous blood withdrawn for biochemical, biomarker and hormonal analysis and baseline renal/liver function for trial eligibility
- Cardiac MRI with gadolinium contrast

8.2.1 Biochemical and hormonal measurements

50mls of venous blood will be collected at three time points during the study - pre-randomisation, study visit 7 (26 weeks) and study visit 9 (52 weeks) for biochemical and hormonal analysis. Where possible, samples will be taken as fasting, early morning samples to account for the diurnal variation in hormonal measurements. Non-fasting samples will be taken in patients with diabetes to avoid any potential risk of hypoglycaemia. All samples will be taken after at least 15 minutes of supine rest. Spot urine collections will be performed at pre-randomisation, 26 weeks and 52 weeks. Samples will be used for the following measurements pertaining to the action of sacubitril/valsartan and remodelling: Brain Natriuretic Peptide (BNP), N-Terminal pro BNP (NT-proBNP), MR-pro Atrial Natriuretic Peptide (MR-proANP), C-terminal ANP, C-type natriuretic peptide (CNP), high sensitivity Troponin-I (hsTnI), Urea and

Electrolytes (U&Es), C-Reactive Protein (CRP), cyclic guanosine monophosphate (cGMP), neprilysin antigen , matrix metalloproteinases (MMPs), Tissue inhibitors of metalloproteinases-1 (TIMP-1), ST2, galectin-3, amino-terminal propeptide of type III procollagen (PIIINP), adrenomedullin (MR-proADM), growth differentiation factor (GDF-15), and endothelin-1. Measurements of glycated haemoglobin A1c (HbA1c) will be made as per standard clinical practice at baseline, 6 months and 12 months for the purposes of screening for, and assessment of diabetic control. Any samples remaining will be stored for analysis of any future relevant biomarkers as they become available and for use in future ethically approved research studies if appropriate.

8.2.2 CMR protocol

All CMR scans will be performed on a single 3.0 Tesla Siemens MAGNETOM Prisma scanner located in the CRF at the QEUH. The MRI scan will include cine MRI, T1 and T2 maps, strain encoded MRI, and contrast enhanced imaging including first pass perfusion at rest, microvascular obstruction and late gadolinium enhancement imaging 10 - 15 min after contrast administration. IV access will be required for contrast administration and a full blood count will be obtained before the scan in order to measure extracellular volume. Specific details of the CMR protocol are detailed in a local Standard Operating Procedure. Patients will be provided with an MRI information sheet prior to their visit (Appendix 2).

8.3 26-week visit

This will comprise of a half-day visit to the QEUH CRF. A number of the baseline investigations will be repeated:

- full physical examination
- spot urine collection
- venepuncture – 50ml blood withdrawn for biochemical and hormonal analysis

Patients will also be asked to complete a short patient global assessment questionnaire (Appendix 3) at this visit.

8.4 52-week visit (- 3 months +6 months)

This will comprise of a half-day visit to the QEUH CRF. A number of the baseline investigations will be repeated:

- full physical examination
- spot urine collection
- venepuncture – 50ml blood withdrawn for biochemical and hormonal analysis
- cardiac MRI with gadolinium contrast will be performed.

Patients will also be asked to complete a short patient global assessment (Appendix 3) questionnaire at this visit.

In response to the COVID-19 pandemic we have allowed an extension of the patients IMP treatment by up to 3 months. During this extended time on IMP we would anticipate following up with patients by telephone every 6 weeks. Patients have the clinical research team contact details should they wish to discuss any aspect of the extended involvement themselves.

At this point a patient's participation in the involvement in the trial will end. They will return any unused study drug. Following their involvement in the trial patients will be converted to an ACE-inhibitor or ARB as per local practice guidelines at an equivalent dose to their final study drug dose level. Prior to commencement of any ACE-inhibitor, patients will require to have a 36-hour washout period following their last dose of study drug in order to reduce the risk of angioedema associated with combined ACE and NEP inhibition.

8.5 Definition of end of trial

The end of the trial is defined as the date of the last patient's 52-week (- 3 months +6 months) study visit plus additional 30 days of event reporting.

9 Assessment, samples and data collection

Trial data will be recorded on trial-specific eCRFs. Only the patient's unique identifier number, date of birth and initials will be recorded on the eCRF in order to identify the patient. Any missing or discrepant data queries will be sent to appropriate research team member(s) to be reviewed. Biochemical samples will be stored in the QUEH Biochemistry department and the BHF Glasgow Cardiovascular Research Centre to be analysed in batches on completion of the trial. Analysis of echocardiograms and CMR imaging will be performed offline.

Table 9-1 Schedule of Assessments

Study Procedure	Screening (>3months post myocardial infarction)	Visit 1 - Randomisation (Week 0)	Visit 2 (Week 1 ± 3 days)	Visit 3 (Week 2± 3 days)	Visit 4 (Week 4± 3 days)	Visit 5 (Week 5± 3 days)	Visit 6 (Week 14 ± 7 days)	Visit 7 (Week 26 ± 7 days)	Visit 8 (Week 39 ± 7 days)	Visit 9 (Week 52 -3 months +6 months)
Review Inclusion/Exclusion Criteria	X									
Echocardiogram	X									
Obtain informed consent	X	X								
Cardiac MRI		X								X
Physical examination		X	X		X			X		X
Medical history	X	X								
Concomitant medications	X	X	X	X	X	X	X	X	X	X
Vital Signs (Blood pressure/Heart rate)	X	X	X	X	X	X	X	X	X	X
12 Lead ECG		X								
Spot urine collection		X						X		X
Venepuncture (Urea and Electrolytes/FBC and LFTs)	X[&]	X	X	X	X	X	X	X	X	X
Venepuncture (Biochemical/hormonal/ biomarker analysis)		X						X		X
Pregnancy testing in WoCBP		X			X		X	X	X	X
Patient global assessment questionnaire								X		X
IMP Dispensing*		X			X		X	X		
Up-titrate IMP#					X					
IMP Administration+		X	X	X	X	X	X	X	X	X
Adverse event reporting			X	X	X	X	X	X	X	X
Study completion										X

[*] Study drug will be introduced at equivalent dose to existing ACE-i/ARB treatment (Dose level 2 or 3) at investigator's discretion

[#]Up-titration not required if patient already on dose level 3 but down titration to dose level 2 permitted in response to COVID-19

[&]If not done in the preceding 6 months

+Emergency IMP dispensing permitted during COVID-19 pandemic

10 Pharmacovigilance

10.1 Definitions

Table 10-1 Pharmacovigilance Definitions

Term	Definition
**Adverse Event (AE)**	Any untoward medical occurrence in a participant to whom a medicinal product has been administered, including occurrences which are not necessarily caused by or related to that product.
**Adverse Reaction (AR)**	An untoward and unintended response in a participant to an investigational medicinal product which is related to any dose administered to that participant.<br>The phrase "response to an investigational medicinal product" means that a causal relationship between a trial medication and an AE is at least a reasonable possibility, i.e. the relationship cannot be ruled out.<br>All cases judged by either the reporting medically qualified professional or the Sponsor as having a reasonable suspected causal relationship to the trial medication qualify as adverse reactions.
**Serious Adverse Event (SAE)**	A serious adverse event is any untoward medical occurrence that:<br>results in death<br>is life-threatening<br>requires inpatient hospitalisation or prolongation of existing hospitalisation<br>results in persistent or significant disability/incapacity<br>consists of a congenital anomaly or birth defect<br>Other events that are considered medically significant may also be considered serious if they jeopardise the participant or require an intervention to prevent one of the above consequences.<br>NOTE: The term "life-threatening" in the definition of "serious" refers to an event in which the participant was at risk of death at the time of the event; it does not refer to an event which hypothetically might have caused death if it were more severe.
**Serious Adverse Reaction (SAR)**	An adverse event that is both serious and, in the opinion of the reporting Investigator, believed with reasonable probability to be due to one of the trial treatments, based on the information provided.
**Suspected Unexpected Serious Adverse Reaction (SUSAR)**	A serious adverse reaction, the nature and severity of which is not consistent with the information about the medicinal product in question set out:

	in the case of a product with a marketing authorisation, in the summary of product characteristics (SmPC) for that product.<br>in the case of any other investigational medicinal product, in the investigator's brochure (IB) relating to the trial in question.

10.2 Recording and reporting of Adverse Events

All AEs occurring during the trial that are observed by the Investigator or reported by the participant should be recorded in the participant's medical records whether or not attributed to trial medication.

Details of the following adverse reactions will be collected within the eCRF at all follow up visits from randomisation until the participants' final visit.

- Clinically relevant hypotension (syncope, dizziness, etc)
- Worsening renal function
- Acute kidney injury
- Hyperkalaemia
- Occurrence of angioedema

The seriousness of each adverse reaction will be collected along with the outcome of the event at the time of the visit i.e. whether the reaction is ongoing at the time of the visit or the participant has recovered.

SAEs will be documented within the eCRF and will be collected from the date of randomisation until 30 days post cessation of trial treatment to allow for reporting of any ongoing adverse reactions associated with the use of the trial drug.

The following events are excluded from the need for expedited reporting:

- Routine treatment or monitoring of the studied indication not associated with any deterioration in condition.
- Treatment which was elective or pre-planned, for a pre-existing condition not associated with any deterioration in condition, e.g. pre-planned hip replacement operation which does not lead to further complications.
- Any admission to hospital or other institution for general care where there was no deterioration in condition.
- Treatment on an emergency, outpatient basis for an event not fulfilling any of the definitions of serious as given above and not resulting in hospital admission.

10.3 Assessment of adverse events

All adverse events must be assessed for seriousness. SAEs occurring between the date of consent and 30 days post treatment must be assessed for causality,

expectedness and severity and notified to the Sponsor. This is the responsibility of the CI or designee.

Seriousness

AEs should be assessed for seriousness as per the definitions in section 10.1; those meeting the criteria for an SAE are subject to expedited reporting to the sponsor.

Assessment of causality

SAEs must be assessed for their causality i.e. does the event have a reasonable causal relationship with the trial medication.

Assessment of expectedness

If an SAE is considered to be related (possibly, probably or definitely) to the study medication, an assessment should be made of the expectedness of the reaction i.e. is the reaction a recognised adverse effect of the medication.

The expectedness of an adverse reaction must be assessed against the Reference Safety Information (RSI) i.e. the list of expected reactions detailed in the Summary of Produce Characteristics (SmPC) for the Investigational Medicinal Product approved during Clinical Trial Authorisation process.

Expected: consistent with the relevant product information documented in the RSI.

Unexpected: not consistent with the relevant product information documented in the RSI.

Assessment of Severity

This should be assessed and described using the following categories:
- Mild-awareness of event but easily tolerated
- Moderate-discomfort enough to cause some interference with usual activity
- Severe-inability to carry out usual activity.

10.4 Reporting to sponsor (Pharmacovigilance Office)

All SAEs arising during the study will be reported by the Principal Investigator (or designee) to the sponsor (PV Office) by completing the trial eCRF as soon as reasonably practicable and in any event within 24 hours of first becoming aware of the event. Any follow up information should also be reported.

In the event that eCRF reporting is not possible, a paper SAE form (CTIMP) can be downloaded from the Glasgow Clinical Trials Unit website: www.glasgowctu.org.

This should be completed and faxed to the PV Office. (Fax No: +44 (0) 141 357 5588). A copy of the complete form should be placed in the Study Site File. If necessary, a verbal report can be given by contacting the PV Office on +44 (0)141

330 4870/ +44 (0)141 330 4744. This must be followed up as soon as possible with a written report.

10.5 Reporting to the MHRA and MREC

All SAEs assigned by the PI or delegate and by the CI (on behalf of the sponsor), as both suspected to be related to IMP-treatment and unexpected will be classified as Suspected Unexpected Serious Adverse Reactions (SUSARs). These will be subject to expedited reporting to the Medicines and Healthcare Products Regulatory Agency (MHRA). The Sponsor Pharmacovigilance office will inform the MHRA, the REC and the Sponsor of SUSARs within the required expedited reporting timescales.

- Fatal or life threatening SUSARs: not later than 7 days after the sponsor had information that the case fulfilled the criteria for a fatal or life threatening SUSAR, and any follow up information within a further 8 days.

- All other SUSARs: not later than 15 days after the sponsor had information that the case fulfilled the criteria for a SUSAR.

10.6 Responsibilities

Chief Investigator (CI) / delegate or independent clinical reviewer:

1. Clinical oversight of the safety of patients participating in the trial, including an ongoing review of the risk / benefit.
2. Using medical judgement in confirming PI's assessment of expectedness of SAEs for 'related' SAEs.
3. Immediate review of all SUSARs.
4. Preparing the clinical sections and final sign off of the Development Safety Update Report (DSUR).

Principal Investigator (PI):

1. Checking for AEs and ARs when participants attend for treatment / follow-up.
2. Using medical judgement in assigning seriousness, severity, causality and expectedness using the Reference Safety Information approved for the trial.
3. Ensuring that all SAEs and SARs (including SUSARs) are recorded and reported to the Sponsor within 24 hours of becoming aware of the event and provide further follow-up information as soon as available.
4. Ensuring that SAEs meeting the definition of SAEs of special interest are marked as such.
5. Using medical judgement in assigning expectedness of SAEs for 'related' SAEs.

Sponsor:

1. Central data collection and verification SAEs, SARs and SUSARs according to the trial protocol.

2. Reporting safety information to the CI, delegate or independent clinical reviewer for the ongoing assessment of the risk / benefit according to the Trial Monitoring Plan.
3. Reporting safety information to the independent oversight committees identified for the trial (Trial Steering Committee (TSC)) according to the Trial Monitoring Plan.
4. Expedited reporting of SUSARs to the Competent Authority (MHRA in UK) and REC within required timelines.
5. Notifying Investigators of SUSARs that occur within the trial.
6. Checking for (annually) and notifying PIs of updates to the Reference Safety Information for the trial.
7. Preparing standard tables and other relevant information for the DSUR in collaboration with the CI and ensuring timely submission to the MHRA and REC.

Trial Steering Committee (TSC):

In accordance with the Trial Terms of Reference for the TSC, periodically reviewing safety data and liaising with the CI and TMG regarding safety issues.

10.7 Pregnancy reporting

Pregnancy is not considered an AE or SAE. However, Principal Investigators will report pregnancy information on any female participant or female partner of a male participant who becomes pregnant while participating in the trial to the sponsor within two weeks of first becoming aware of the pregnancy. This report should be provided to the PV office on the Pregnancy Notification Form provide by the sponsor (on www.glasgowctu.org). The subject will also be followed to determine the outcome of the pregnancy. Information on the status of the mother and child will be forwarded by the PI to the PV Office

10.8 Development safety update reports

A Development Safety Update Report (DSUR) will be submitted once a year, or on request, to MHRA and REC until the trial is declared ended. The report will be submitted within 60 days of the anniversary of the issue of the Clinical Trials Authorisation for the trial. The report will be prepared by the sponsor (PV Office) in liaison with the CI and submitted by the sponsor (PV Office).

11 Statistics and data analysis plan

11.1 Sample size

A sample size of 100 patients is proposed. This is based on the calculation that 45 patients per treatment group provides >90% power (α level = 0.05) to detect a difference of 6mL/m^2 in LVESVI (standard deviation = 7.8mL/m^2) and accounting for a discontinuation rate of 10% (lost to follow up, development of heart failure or

death).[37] A 6mL/m^2 difference in LVESVI represents a minimally important difference.

11.2 Planned recruitment rate

Recruitment of 100 patients in 18 months requires a monthly recruitment rate of 5.6 patients/month or 1.4 patients/week. We have previously recruited patients (n=100) in a study with similar inclusion criteria within the time-frame of 12 months.[38] Data collected from a pharmacy led “post MI clinic” for patients with LVSD following an MI report a yearly incidence of around 90 patients with an LVEF ≤ 40% and without any evidence of clinical HF (NYHA I). We plan to use the database from these clinics to identify suitable patients as well as recruiting patients newly referred to the clinic following a MI. Recent restructuring of the health services in Glasgow has resulted in the amalgamation of 3 hospitals (Western Infirmary, Southern General and the Victoria Infirmary) into a single site at the Queen Elizabeth University Hospital (QEUH). We plan to base recruitment at this site given its large catchment population of 560,000. We will also recruit patients from QEUH, GRI, RAH, New Victoria Hospital, West Glasgow Ambulatory Care Hospital, Inverclyde Hospital, Vale of Leven Hospital, Wishaw General Hospital, Hairmyres Hospital and Monklands General Hospital via post-myocardial infarction outpatient clinics and general cardiology clinics.

11.3 Statistical analysis

The study will have a comprehensive Statistical Analysis Plan, which will be authored by the Trial Statistician and agreed by the Investigators before the final unblinded analysis. Analysis will be done on an intention-to-treat basis. The Robertson Centre for Biostatistics, functional unit of the Glasgow Clinical Trials Unit, a fully registered UK CRN Clinical Trials Unit, will manage and analyse trial data.

The primary analysis will compare LVESVI at 12 months between randomised groups using a linear regression model, adjusted for baseline LVESVI, and whether or not taking diuretics at baseline. Similar methods will be applied to secondary outcomes. Safety data will be summarised without formal statistical comparison.

12 Data handling

12.1 Randomisation

Randomisation will be in a 1:1 ratio and stratified by LVESVI ≤45ml/m^2 / >45ml/m^2 as measured by baseline CMR, and whether or not the patient is being prescribed diuretics at baseline, with the randomisation schedule computer generated by the method of randomised permuted blocks, with random block lengths of 4 and 6. The allocation for each patient will not be known to researchers until the point of randomisation. The full allocation sequence will be stored in a secure area of the Robertson Centre for Biostatistics network, with access restricted to those responsible for maintenance of the randomisation system. Study statisticians will not have access to the allocation schedule until the database has been locked, and all

analysis programs have been validated according to a prespecified statistical validation plan.

12.2 Case Report Forms

A study specific electronic case report form (eCRF) will be used to collect study data. It is the investigator's responsibility to ensure completion and to review and approve all data captured in the eCRF. All data handling procedures will be detailed in a Study Specific Data Management Plan. Data will be validated at the point of entry into the eCRF and at regular intervals during the study. Data discrepancies will be flagged to the investigators and any data changes will be recorded in order to maintain a complete audit trail (reason for change, date change made, who made change).

12.3 Record Retention

To enable evaluations and/or audits from regulatory authorities, the investigator agrees to keep records, including the identity of all participating subjects (sufficient information to link records), all original signed informed consent forms, serious adverse event forms, source documents, and detailed records of treatment disposition in accordance with EU GCP, local regulations, or as specified in the Clinical Study Agreement, whichever is longer. Data will be retained for a minimum of 25 years or for the time-period required to meet regulatory requirements defined for this trial.

13 Trial management

13.1 Trial steering committee (TSC)

A TSC will oversee the running of the trial and ensure that it is being conducted in accordance with the principles of GCP and the relevant regulations.

14 Study monitoring and auditing

Study monitoring visits will be conducted by NHS Greater Glasgow and Clyde (GG&C) Monitors. The level of monitoring will be based on the outcome of the completed monitoring risk assessment, and will be clearly documented in the monitoring plan which will be approved by the NHS GG&C Research Governance Manager / Academic Lead Clinical Trial Monitor. As standard, monitoring visit(s) will cover site file review, review of Informed Consent Forms (ICFs), Source Data Verification (SDV) and Serious Adverse Event (SAE) review as per monitoring plan objectives.

15 Ethical considerations

The study will be carried out in accordance with the World Medical Association Declaration of Helsinki (1964) and its revisions (Tokyo [1975], Venice [1983], Hong Kong [1989], South Africa [1996] Edinburgh [2000], Seoul [2008] and Fortaleza

[2013]). Favourable ethical opinion will be sought from the East of Scotland Research Ethics Committee (REC) before patients are entered into this clinical trial. Patients will only be allowed to enter the study once wither they have provided written informed consent. The CI will be responsible for updating the REC of any new information related to the study. The participant will remain free to withdraw from the study at any time without giving reasons and without prejudicing his/her further treatment.

16 Statement of indemnity

The trial is sponsored by NHS Greater Glasgow & Clyde and the University of Glasgow. The sponsor will be liable for negligent harm caused by the design of the trial. NHS indemnity is provided under the Clinical Negligence and Other Risks Indemnity Scheme (CNORIS). The NHS has a duty of care to patients treated, whether or not the patient is taking part in a clinical study, and the NHS remains liable for clinical negligence and other negligent harm to patients under its duty of care.

17 Funding

This trial is funded by the British Heart Foundation (PG/17/23/32850) and trial medications are supplied by the drug company Novartis who produce both valsartan (the standard treatment) sacubitril/valsartan (the experimental treatment).

18 Sponsor responsibilities

The sponsor of this clinical study is NHS Greater Glasgow and Clyde/University of Glasgow. Sponsor responsibilities undertaken by NHS Greater Glasgow and Clyde/University of Glasgow will be as defined in the Research Governance Framework for Health and Community Care (Second edition, February 2006).

19 Dissemination of data

The study will be assigned an International Standard Randomised Controlled Trial Number (ISRCTN) and will be registered on the clinicaltrials.gov website before participant recruitment commences.

Once the study has completed and data has been analysed the results will be submitted for publication in a peer-reviewed scientific journal and presented at a major conference. The research team will provide a lay-summary of the results to all participants where appropriate and inform the patients general practitioner of the results of the trial. Data will be made available in an anonymised summary for regulatory purposes or on the request of the funder/Sponsor.

20 References

1. Keeley EC, Boura JA, Grines CL. Primary angioplasty versus intravenous thrombolytic therapy for acute myocardial infarction: a quantitative review of 23 randomised trials. *Lancet* 2003;**361**:13–20.
2. Indications for fibrinolytic therapy in suspected acute myocardial infarction: collaborative overview of early mortality and major morbidity results from all randomised trials of more than 1000 patients. Fibrinolytic Therapy Trialists' (FTT) Collaborative. *Lancet* 1994;**343**:311–322.
3. British Heart Foundation. Cardiovascular Disease Statistics, 2015. https://www.bhf.org.uk/publications/statistics/cvd-stats-2015 (18 February 2016)
4. Velagaleti RS, Pencina MJ, Murabito JM, Wang TJ, Parikh NI, D'Agostino RB, Levy D, Kannel WB, Vasan RS. Long-term trends in the incidence of heart failure after myocardial infarction. *Circulation* 2008;**118**:2057–2062.
5. Sulo G, Igland J, Vollset SE, Nygård O, Ebbing M, Sulo E, Egeland GM, Tell GS. Heart Failure Complicating Acute Myocardial Infarction; Burden and Timing of Occurrence: A Nation-wide Analysis Including 86771 Patients From the Cardiovascular Disease in Norway (CVDNOR) Project. *J Am Heart Assoc* 2016;**5**:e002667. doi: 10.1161/JAHA.115.002667.
6. Pfeffer MA, Braunwald E, Moyé LA, Basta L, Brown EJ, Cuddy TE, Davis BR, Geltman EM, Goldman S, Flaker GC. Effect of captopril on mortality and morbidity in patients with left ventricular dysfunction after myocardial infarction. Results of the survival and ventricular enlargement trial. The SAVE Investigators. *N Engl J Med* 1992;**327**:669–677.
7. The Acute Infarction Ramipril Efficacy (AIRE) Study Investigators. Effect of ramipril on mortality and morbidity of survivors of acute myocardial infarction with clinical evidence of heart failure. *Lancet* 1993;**342**:821–828.
8. Køber L, Torp-Pedersen C, Carlsen JE, Bagger H, Eliasen P, Lyngborg K, Videbaek J, Cole DS, Auclert L, Pauly NC. A clinical trial of the angiotensin-converting-enzyme inhibitor trandolapril in patients with left ventricular dysfunction after myocardial infarction. Trandolapril Cardiac Evaluation (TRACE) Study Group. *N Engl J Med* 1995;**333**:1670–1676.
9. Pfeffer MA, McMurray JJ V, Velazquez EJ, Rouleau J-L, Køber L, Maggioni AP, Solomon SD, Swedberg K, Werf F Van de, White H, Leimberger JD, Henis M, Edwards S, Zelenkofske S, Sellers MA, Califf RM, Valsartan in Acute Myocardial Infarction Trial Investigators. Valsartan, captopril, or both in myocardial infarction complicated by heart failure, left ventricular dysfunction, or both. *N Engl J Med* 2003;**349**:1893–1906.
10. The CONSENSUS Trial Study Group. Effects of enalapril on mortality in severe congestive heart failure. Results of the Cooperative North Scandinavian Enalapril Survival Study (CONSENSUS). *N Engl J Med* 1987;**316**:1429–1435.
11. The SOLVD Investigators. Effect of enalapril on survival in patients with reduced left ventricular ejection fractions and congestive heart failure. *N Engl J Med* 1991;**325**:293–302.
12. Cohn JN, Tognoni G. A randomized trial of the angiotensin-receptor blocker valsartan in chronic heart failure. *N Engl J Med* 2001;**345**:1667–1675.
13. Granger CB, McMurray JJ V, Yusuf S, Held P, Michelson EL, Olofsson B, Ostergren J, Pfeffer MA, Swedberg K. Effects of candesartan in patients with

chronic heart failure and reduced left-ventricular systolic function intolerant to angiotensin-converting-enzyme inhibitors: the CHARM-Alternative trial. *Lancet* 2003;**362**:772–776.
14. Dargie HJ, Lechat P. The Cardiac Insufficiency Bisoprolol Study II (CIBIS-II): A randomised trial. *Lancet* 1999;**353**:9–13.
15. MERIT-HF Study Group. Effect of metoprolol CR/XL in chronic heart failure: Metoprolol CR/XL Randomised Intervention Trial in Congestive Heart Failure (MERIT-HF). *Lancet* 1999;**353**:2001–2007.
16. Packer M, Fowler MB, Roecker EB, Coats AJS, Katus H a., Krum H, Mohacsi P, Rouleau JL, Tendera M, Staiger C, Holcslaw TL, Amann-Zalan I, DeMets DL. Effect of carvedilol on the morbidity of patients with severe chronic heart failure: Results of the carvedilol prospective randomized cumulative survival (COPERNICUS) study. *Circulation* 2002;**106**:2194–2199.
17. Dargie HJ. Effect of carvedilol on outcome after myocardial infarction in patients with left-ventricular dysfunction: the CAPRICORN randomised trial. *Lancet* 2001;**357**:1385–1390.
18. Pitt B, Remme W, Zannad F, Neaton J, Martinez F, Roniker B, Bittman R, Hurley S, Kleiman J, Gatlin M. Eplerenone, a selective aldosterone blocker, in patients with left ventricular dysfunction after myocardial infarction. *N Engl J Med* 2003;**348**:1309–1321.
19. Zannad F, McMurray JJ V, Krum H, Veldhuisen DJ van, Swedberg K, Shi H, Vincent J, Pocock SJ, Pitt B. Eplerenone in patients with systolic heart failure and mild symptoms. *N Engl J Med* 2011;**364**:11–21.
20. The SOLVD Investigators. Effect of enalapril on mortality and the development of heart failure in asymptomatic patients with reduced left ventricular ejection fractions. *N Engl J Med* 1992;**327**:685–691.
21. St John Sutton M, Pfeffer MA, Plappert T, Rouleau JL, Moyé LA, Dagenais GR, Lamas GA, Klein M, Sussex B, Goldman S. Quantitative two-dimensional echocardiographic measurements are major predictors of adverse cardiovascular events after acute myocardial infarction. The protective effects of captopril. *Circulation* 1994;**89**:68–75.
22. Solomon SD, Skali H, Anavekar NS, Bourgoun M, Barvik S, Ghali JK, Warnica JW, Khrakovskaya M, Arnold JMO, Schwartz Y, Velazquez EJ, Califf RM, McMurray J V., Pfeffer MA. Changes in ventricular size and function in patients treated with valsartan, captopril, or both after myocardial infarction. *Circulation* 2005;**111**:3411–3419.
23. Woods RL. Cardioprotective functions of atrial natriuretic peptide and B-type natriuretic peptide: a brief review. *Clin Exp Pharmacol Physiol* 2004;**31**:791–794.
24. McMurray JJV V, Packer M, Desai AS, Gong J, Lefkowitz MP, Rizkala AR, Rouleau JL, Shi VC, Solomon SD, Swedberg K, Zile MR, PARADIGM-HF Investigators and Committees. Angiotensin–Neprilysin Inhibition versus Enalapril in Heart Failure. *N Engl J Med* 2014;**371**:993–1004.
25. Ponikowski P Voors A Anker S Bueno H Cleland J et. al. 2016 ESC Guidelines for the diagnosis and treatment of acute and chronic heart failure: The Task Force for the diagnosis and treatment of acute and chronic heart failure of the European Society of Cardiology (ESC). *Eur Heart J* 2016;**37**:2129–2200.
26. Lueder TG von, Wang BH, Kompa AR, Huang L, Webb R, Jordaan P, Atar D, Krum H. Angiotensin Receptor Neprilysin Inhibitor LCZ696 Attenuates Cardiac Remodeling and Dysfunction After Myocardial Infarction by Reducing Cardiac

Fibrosis and Hypertrophy. *Circ Heart Fail* 2015;**8**:71–78.
27. Prospective ARNI vs ACE Inhibitor Trial to DetermIne Superiority in Reducing Heart Failure Events After MI (PARADISE-MI). https://clinicaltrials.gov/ct2/show/NCT02924727 (21 November 2016)
28. ACE Inhibitor Myocardial Infarction Collaborative Group: M.G. Franzosi, E. Santoro, G. Zuanetti C, Baigent, R. Collins, M. Flather, J. Kjekshus RL, L.S. Liu, A.P. Maggioni, P. Sleight, K. Swedberg GT, Yusuf S. Clinical Investigation and Reports Indications for ACE Inhibitors in the Early Treatment of Acute Myocardial Infarction. 1998;2202–2212.
29. Konstam MA, Kronenberg MW, Rousseau MF, Udelson JE, Melin J, Stewart D, Dolan N, Edens TR, Ahn S, Kinan D, et al., Howe DM, Kilcoyne L, Metherall J, Benedict C, Yusuf S, Pouleur H. Effects of the angiotensin converting enzyme inhibitor enalapril on the long-term progression of left ventricular dilatation in patients with asymptomatic systolic dysfunction. *Circulation* 1993;**88**:2277–2283.
30. Gu J, Noe A, Chandra P, Al-Fayoumi S, Ligueros-Saylan M, Sarangapani R, Maahs S, Ksander G, Rigel DF, Jeng AY, Lin TH, Zheng W, Dole WP. Pharmacokinetics and pharmacodynamics of LCZ696, a novel dual-acting angiotensin receptor-neprilysin inhibitor (ARNi). *J Clin Pharmacol* 2010;**50**:401–414.
31. Pennell DJ. Cardiovascular magnetic resonance. *Circulation* 2010;**121**:692–705.
32. White HD, Norris RM, Brown MA, Brandt PW, Whitlock RM, Wild CJ. Left ventricular end-systolic volume as the major determinant of survival after recovery from myocardial infarction. *Circulation* 1987;**76**:44–51.
33. Migrino RQ, Young JB, Ellis SG, White HD, Lundergan CF, Miller DP, Granger CB, Ross AM, Califf RM, Topol EJ. End-systolic volume index at 90 to 180 minutes into reperfusion therapy for acute myocardial infarction is a strong predictor of early and late mortality. The Global Utilization of Streptokinase and t-PA for Occluded Coronary Arteries (GUSTO)-I Angiograph. *Circulation* 1997;**96**:116–121.
34. Steg PG, James SK, Atar D, Badano LP, Blömstrom-Lundqvist C, Borger MA, Mario C Di, Dickstein K, Ducrocq G, Fernandez-Aviles F, Gershlick AH, Giannuzzi P, Halvorsen S, Huber K, Juni P, Kastrati A, Knuuti J, Lenzen MJ, Mahaffey KW, Valgimigli M, 't Hof A van, Widimsky P, Zahger D. ESC Guidelines for the management of acute myocardial infarction in patients presenting with ST-segment elevation. *Eur Heart J* 2012;**33**:2569–2619.
35. Thygesen K, Alpert JS, Jaffe AS, Simoons ML, Chaitman BR, White HD, Katus HA, Lindahl B, Morrow DA, Clemmensen PM, Johanson P, Hod H, Underwood R, Bax JJ, Bonow RO, Pinto F, Gibbons RJ, Fox KA, Atar D, Newby LK, Galvani M, Hamm CW, Uretsky BF, Steg PG, Wijns W, Bassand J-P, Menasché P, Ravkilde J, Ohman EM, Antman EM, et al. Third universal definition of myocardial infarction. *Circulation* 2012;**126**:2020–2035.
36. Lang RM, Badano LP, Mor-Avi V, Afilalo J, Armstrong A, Ernande L, Flachskampf FA, Foster E, Goldstein SA, Kuznetsova T, Lancellotti P, Muraru D, Picard MH, Rietzschel ER, Rudski L, Spencer KT, Tsang W, Voigt J-U. Recommendations for cardiac chamber quantification by echocardiography in adults: an update from the American Society of Echocardiography and the European Association of Cardiovascular Imaging. *Eur Heart J Cardiovasc Imaging* 2015;**16**:233–270.

37. Heydari B, Abdullah S, Pottala J V., Shah R, Abbasi S, Mandry D, Francis SA, Lumish H, Ghoshhajra BB, Hoffmann U, Appelbaum E, Feng JH, Blankstein R, Steigner M, McConnell JP, Harris W, Antman EM, Jerosch-Herold M, Kwong RY, Mozaffarian D, Rimm E, Caterina R De, Investigators G-P, Group OS, Chan W, Duffy S, White D, Gao X, Du X, Ellims A, et al. Effect of Omega-3 Acid Ethyl Esters on Left Ventricular Remodeling After Acute Myocardial InfarctionClinical Perspective. *Circulation* 2016;**134**:1885–1899.
38. Weir R a P, Mark PB, Petrie CJ, Clements S, Steedman T, Ford I, Ng LL, Squire IB, Wagner GS, McMurray JJ V, Dargie HJ. Left ventricular remodeling after acute myocardial infarction: Does eplerenone have an effect? *Am Heart J* Mosby, Inc.; 2009;**157**:1088–1096.

Appendix 1: Clinically notable laboratory values

Clinically notable laboratory abnormalities for selected tests based on a percent change from baseline:

Haematology

Hematocrit >50% increase, >20% decrease
Haemoglobin >50% increase, >20% decrease
Platelet count >75% increase, >50% decrease
RBC Count >50% increase, >20% decrease
WBC count >50% increase, >50% decrease

Biochemistry

Alkaline phosphatase >100% increase
ALT >150% increase
AST >150% increase
BUN >50% increase
Chloride >10% increase, >10% decrease
Creatinine >50% increase
Potassium >20% increase, >20% decrease
Total bilirubin >100% increase

Appendix 2: MRI Information Leaflet

Information about your<br>**MRI (Magnetic Resonance Imaging) Scan**	**NHS**<br>Greater Glasgow and Clyde

Your doctor has referred you for a MRI (Magnetic Resonance Imaging) scan.

What is MRI?
A MRI scan uses a strong magnetic field and computer to build up a picture of the inside of the body.

Before your MRI Scan
For your own safety and to avoid a wasted journey it is essential you fill in the enclosed MRI Safety Checklist Questionnaire. Please bring the completed questionnaire with you. If you have any questions about being suitable for a MRI scan please contact the department (see contact details on the back page).

You cannot have a MRI scan if you have any of the following:

- Cardiac Pacemaker
- Cochlear (ear) Implant
- A metal aneurysm clip in the brain
- Metallic fragments in the eye
- Certain Mechanical Implants.

Diabetes
If you are diabetic please continue to take any medication as usual.

Female Patients
If you are pregnant or think you might be pregnant please contact the department (see contact details on the back page).

If you are breastfeeding please contact the department as you may need an injection of contrast agent (dye) which means you will be unable to breastfeed for a period of time after your MRI, usually 24 hours.

Do I need to prepare?
No. There is no special preparation.

You can eat and drink as normal and take any prescribed medicines, unless your doctor tells you otherwise.

We may ask you to change into a hospital gown. We may ask you to remove dentures, jewellery, hearing aids, hairpins and eye makeup.

Please do not bring any valuables to your scan.

What happens during the scan?

We will ask you to lie on a comfortable couch, which we then move into the magnet.

We will ask you to keep your body perfectly still.

You will not experience any discomfort, but during the scan you may hear some buzzing or tapping noises, which are perfectly harmless.

The radiographer operating the scanner will be able to see and hear you throughout the scan. You will also have a buzzer which you can press if you have any problems and the radiographer will talk to you.

You may bring a CD to listen to during the scan.

How long does the scan last?

The scan may take between 30 minutes and 2 hours. You can leave immediately after the MRI Scan.

When will I know the results?

A radiologist (a specialised doctor) will review your scans and send the results to the person who referred you for the test. Your referrer will make arrangements to tell you the result.

Any other questions?

Please ask the radiographer in the MRI Department before or during your scan.

Your appointment

If you can't make this appointment please tell us as soon as possible – we can arrange another appointment and give your appointment to another patient.

Please be on time when you come for your scan.

Call us if you need advice.

Contact Centre Telephone Number

0141 347 8379
www.nhsgg.org.uk

Equalities In Health

Review Date: July 2018

MI • 290675

Appendix 3: Patient global assessment questionnaire
