# Supplementary material for: Effect of Neprilysin Inhibition on Alzheimer Disease Plasma Biomarkers: A Secondary Analysis of a Randomized Clinical Trial
Source: JAMA Neurol. 2023 Dec 18;81(2):197–200. doi: 10.1001/jamaneurol.2023.4719 (PMC10728797; doi:10.1001/jamaneurol.2023.4719)
Supplement: Supplement 2. — eMethods eFigure. CONSORT Flow Diagram [file jamaneurol-e234719-s002.pdf]

Supplemental Online Content

Brum WS, Docherty KF, Ashton NJ, et al. Effect of neprilysin inhibition on Alzheimer disease plasma biomarkers: an exploratory analysis of a randomized clinical trial. *JAMA Neurol*. Published online December 18, 2023. doi:10.1001/jamaneurol.2023.4719

eMethods

eFigure. CONSORT Flow Diagram

This supplemental material has been provided by the authors to give readers additional information about their work.

© 2023 American Medical Association. All rights reserved.

eMethods

Patients and study design

This study was an exploratory, post hoc, biomarker analysis of a previously published clinical trial. The trial protocol (Supplement 1) and any substantial amendments were approved by the East of Scotland Research Ethics Committee. All patients provided written consent. The trial is registered (http://www.clinicaltrials.gov. Unique identifier: NCT03552575).

Patients and study design

This analysis was based on a prospective, multicenter, double-blind, placebo-controlled, randomized clinical trial evaluating the effect of sacubitril/valsartan (target dose 97/103mg twice daily) compared with valsartan (target dose 160mg twice daily) on ventricular remodeling in cognitively unimpaired individuals with asymptomatic left ventricular systolic dysfunction at least 3 months following myocardial infarction. The trial recruitment occurred across 7 sites in the National Health Service Greater Glasgow and Clyde Health Board between July 2018 and June 2019, with follow-up visits completed in June 2020.

Patients above 18 years of age were eligible for recruitment if they had a left ventricular ejection fraction ≤40% at least three months after an acute myocardial infarction without any symptoms of HF (*i.e.*, New York Heart Association class I), had a systolic blood pressure ≥100 mm Hg, were taking a minimum or greater dose of angiotensin-converting enzyme inhibitors/angiotensin-receptor blockers (ramipril 2.5mg twice daily or equivalent) or were able to tolerate such a dose, and if they were treated with a β-blocker, unless contraindicated or intolerant. Patients were considered ineligible if they had persistent or permanent atrial fibrillation, a serum potassium level of >5.2 mmol/L, or an estimated glomerular filtration rate of <30 mL/min per 1.73 m^2. Further details on inclusion and exclusion criteria can be found in the trial's primary publication. All patients were cognitively unimpaired to the extent of being able to independently understand and follow the study protocol as assessed by the lead trial investigator and remained so through to the end-of-trial visit.

© 2023 American Medical Association. All rights reserved.

Participants were 1:1 randomized to receive either sacubitril/valsartan (target dose, 97/103 mg twice daily) or valsartan (target dose, 160 mg twice daily), by using an interactive web response system. Randomization occurred using randomized permuted blocks of length 4 and 6 (at random), and was stratified by baseline left ventricular end-systolic-volume index, as measured by magnetic resonance imaging, and by use of diuretics at baseline. Patients were randomized to receive assigned therapy for 52 weeks and to remain on the same study drug until the end-of-trial visit, and they attended 10 visits in total (1, 2, 4, 5, 14, 26, 39, and 52 weeks). Plasma samples were collected at baseline, 26, and 52 weeks. Uptitration of sacubitril/valsartan or valsartan to the target dose was attempted during the first 4 weeks after randomization, depending on safety criteria. Patients' blood pressure and renal function were monitored at all visits. All patients and trial staff were blinded to treatment allocation. Further methodological details can be found in the trial's design and results publications.

Blood biomarker analyses

For this study, plasma samples collected in ethylenediaminetetraacetic (EDTA) tubes were used. Plasma Aβ42, Aβ40, Aβ42/Aβ40, GFAP, and NfL were quantified with a Single molecule array (Simoa) HD-X platform (Quanterix, Billerica, MS, USA), with the commercial research kit Neurology 4-Plex-E (Quanterix). Plasma p-tau181 was measured using a validated *in-house* assay (University of Gothenburg), also with a Simoa HD-X equipment. Plasma p-tau217 was quantified using a validated research immunoassay developed by Lilly Research Laboratories (Indianapolis, IN, USA), on a Mesoscale Discovery platform. Simoa measurements were done at the Clinical Neurochemistry Laboratory (University of Gothenburg, Sweden), and plasma p-tau217 analyses were carried out at the Clinical Memory Research Unit (Lund University, Sweden). All scientists involved in biomarker measurements were blinded to sample information. Due to the volume of plasma collected, analyses of plasma Aβ and p-tau217 were prioritized, making a few follow-up p-tau181 measurements unavailable, as shown in the number at risk table in the main results figure (Fig. 1). The order of analyzed samples was randomized,

© 2023 American Medical Association. All rights reserved.

with all biomarker analyses carried out in duplicate and with longitudinal samples from the same patient being always analyzed in the same run.

Statistical analyses

Plasma Aβ42, Aβ40, Aβ42/Aβ40, p-tau181, p-tau217, GFAP and NfL values were log10-transformed to normalize the data, and change from baseline was computed based on these values, as recently described in an exploratory analysis of an anti-Aβ AD clinical trial using the same biomarkers as used here. Mixed models for repeated measures were used to compare change from baseline for each biomarker between treatment groups at 26 and 52 weeks. All models included categorical fixed effects for treatment group, visit, and treatment group-by-visit interaction. The models were adjusted for each biomarker's baseline value and age and were fitted using an unstructured covariance matrix. The adjusted least-square mean difference and standard errors in biomarker change from baseline are reported individually for each treatment at each visit, and the between-group difference from baseline to 26- and 52 weeks is reported as a relative change calculated by back-transformation of the coefficients. For visualizing changes in plasma Aβ biomarkers, we also report boxplots and histograms of percent change from baseline to the end-of-trial visit for each group. Statistical significance was set at a two-sided p-value of 0.05. All analyses were exploratory and not corrected for multiple testing. Statistical analyses were performed in R version 4.1.1 (www.r-project.org).

© 2023 American Medical Association. All rights reserved.

eFigure 1 CONSORT Flow Diagram

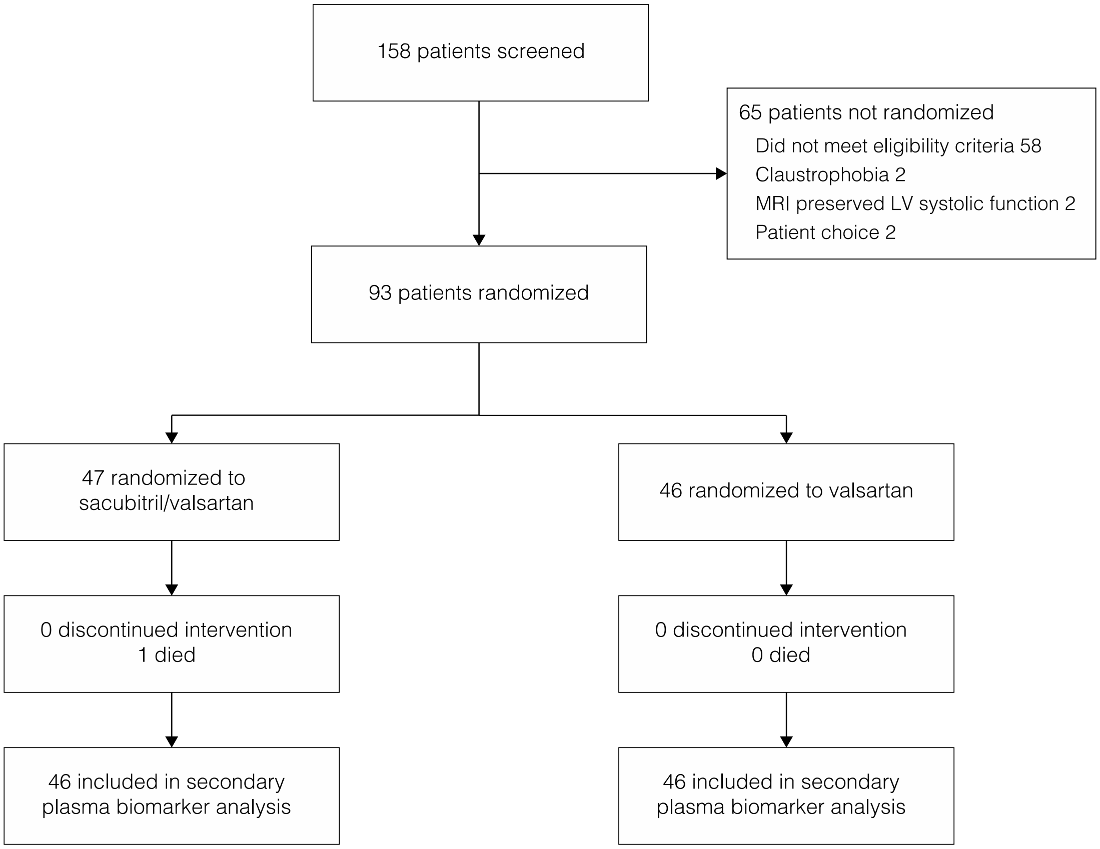

CONSORT diagram illustrating the flowchart of pacient inclusion in this exploratory analysis.

© 2023 American Medical Association. All rights reserved.
