# Supplementary material for: Effect of Neprilysin Inhibition on Alzheimer Disease Plasma Biomarkers: A Secondary Analysis of a Randomized Clinical Trial
Source: JAMA Neurol. 2023 Dec 18;81(2):197–200. doi: 10.1001/jamaneurol.2023.4719 (PMC10728797; doi:10.1001/jamaneurol.2023.4719)
Supplement: Supplement 3. — Data Sharing Statement [file jamaneurol-e234719-s003.pdf]

Data Sharing Statement

Brum. Effect of Neprilysin Inhibition on Alzheimer Disease Plasma Biomarkers. *JAMA Neurol*.
Published December 18, 2023. doi:10.1001/jamaneurol.2023.4719

Data
Data available: No
